# Ants of the Genus *Solenopsis* Westwood 1840 (Hymenoptera: Formicidae) in the Arabian Peninsula with Description of a New Species, *Solenopsis elhawagryi*

**DOI:** 10.1371/journal.pone.0049485

**Published:** 2012-11-30

**Authors:** Mostafa R. Sharaf, Abdulrahman S. Aldawood

**Affiliations:** Plant Protection Department, College of Food and Agriculture Sciences, King Saud University, Riyadh, Kingdom of Saudi Arabia; University of Arizona, United States of America

## Abstract

Ants of the genus *Solenopsis* Westwood in the Arabian Peninsula are revised. Six species are treated: *Solenopsis elhawagryi* Sharaf & Aldawood sp. n., *S. geminata* (Fabricius, 1804), *S. omana* Collingwood & Agosti, 1996, *S. saudiensis* Sharaf & Aldawood, 2011, *S. sumara* Collingwood & Agosti, 1996, and *S. zingibara* Collingwood & Agosti, 1996. *Solenopsis elhawagryi* is described from Beljorashi Governorate, Al Baha Province, Saudi Arabia, based on worker castes and the queen with notes on this species biology and ecology. *Solenopsis sumara* workers are redescribed and illustrated for the first time and a lectotype is designated. An identification key to the Arabian and Egyptian species is provided with scanning electron micrographs to facilitate species recognition.

## Introduction

This is the second contribution to the revision of the ant genera of the Arabian Peninsula (referred to hereafter as “Arabia”). The first paper treated the genus *Plagiolepis* [1]. This revision concerns the genus *Solenopsis* as defined by Westwood [2] with the type species *S. mandibularis* Westwood. Often known as thief ants, globally this is one of the largest genera in the subfamily Myrmicinae, with more than 285 recognized taxa [3] distributed in tropical and warm temperate regions [4].

The *Solenopsis* character states follow the definitions of Ettershank [5] and Bolton [6]: members of the genus are monomorphic or polymorphic. The mandibles of *Solenopsis* species are tri or quadridentate; a palp formula of 2, 2 or 1, 2; the clypeus longitudinally bicarinate, with the median area sharply elevated and deeply inserted between the closely approximated frontal lobes; the anterior clypeal margin with a single long median seta; antennae ten-segmented with a two-segmented club; propodeum unarmed; petiole always distinctly pedunculate, node high and rounded.

Despite their abundance, few revisionary treatments have included *Solenopsis*. A global study of taxa related to *Solenopsis* and *Pheidologeton* Mayr listed 257 species [5]. New World species of the *Solenopsis geminata*-group were reviewed by Trager [7], who supported the synonymy of *Bisolenopsis* Kusnezov, *Synsolenopsis* Forel, *Paranamyrma* Kusnezov, and *Labauchena* Santschi, with *Solenopsis*. Recently, a comprehensive review of North American thief ants recognized eighty-three taxa including eleven new species [8].

The scarcity of published knowledge may be due to the difficult taxonomy of the genus when compared to other Formicidae and the small size of workers. Because of their small size, pale coloration, and cryptic habitats, the Arabian species may well have been overlooked by collectors. Perhaps the best expression of this situation is the statement of Creighton [9]: “The student of North American ants may count himself fortunate that so few species of this difficult genus occur in our latitudes. He/she is thus saved from the task of trying to distinguish the many tropical species whose worker caste shows an astonishing and baffling convergence.”

The first and sole paper which treats Arabian *Solenopsis* is that of Collingwood & Agosti [10]. The genus was reported for the first time from Arabia where three new species are described including, *Solenopsis omana* from Oman and UAE, *Solenopsis sumara* and *S. zingibara* from Yemen. The descriptions, however, are relatively brief and only *S. omana* was illustrated.

A year later, the invasive pest, *S. geminata* (Fabricius) was recorded from UAE, where it was causing irritating nuisance to race horses and camels [11]. Recently, the genus was reported in Saudi Arabia (Riyadh), with a further new species, *S. saudiensis*, described and provided with ecological and biological notes [12]. The authors presented the first key to the known four Arabian species together with the four known Egyptian species, *S. cooperi* Donisthorpe, *S. lou* Forel, *S. occipitalis* Santschi, and *S. kochi* Finzi [13]. *Solenopsis geminata* was not included.

Here, the *Solenopsis* fauna of Arabia is reviewed. *Solenopsis elhawagryi* sp. n. is described and illustrated based on worker and queen castes and notes on its natural history are provided. *Solenopsis sumara* is redescribed, comprehensively measured, and illustrated for the first time, with designation of a lectotype. A key to the six recorded Arabian species with the four Egyptian species is presented to facilitate species regional recognition, although the type specimens of *S. zingibara* appear to be lost [Guy Knight, WMLC, Daniel Burkhardt, Isabelle Pfander, NHMB, personal communications] and the taxonomic treatment of this species is based on the original description.

## Materials and Methods

### Measurements and indices

TL = Total Length; the outstretched length of the ant from the mandibular apex to the metasomal apex.

HW = Head Width; the maximum width of the head behind eyes in full face view.

HL = Head Length; the maximum length of the head, excluding the mandibles.

CI = Cephalic Index (HW×100/HL).

SL = Scape Length, excluding basal neck.

SI = Scape Index (SL×100/HW).

EL = Eye Length; the maximum diameter of the eye.

ML = Mesosoma Length; the length of the mesosoma in lateral view, from the point at which the pronotum meets the cervical shield to the posterior base of the propodeal lobes or teeth.

PRW = Pronotal width, maximum width in dorsal view.

PL = Petiole Length; the maximum length measured in dorsal view, from the anterior margin to the posterior margin.

PW = Petiole Width; maximum width measured in dorsal view.

PPL = Postpetiole Length; maximum length measured in dorsal view.

PPW = Postpetiole Width; maximum width measured in dorsal view.

All measurements are in millimeters and follow the standard measurements [14].

Specimens of *Solenopsis* were examined from the following collections:

**BMNH** Natural History Museum, London, United Kingdom.

**CACC** Cedric A. Collingwood Collection.

**CASC** California Academy of Science Collection, San Francisco, California, USA.

**KSMA** King Saud Museum of Arthropods, King Saud University, Riyadh, Kingdom of Saudi Arabia.

**MHNG** Muséum ďHistoire Naturelle, Geneva, Switzerland.

**NHMB** Naturhistorisches Museum, Basel, Switzerland.

**TAUI** Tel Aviv University Entomological Collection, Israel.

**WMLC** World Museum Liverpool, Liverpool, United Kingdom.

**CACC** Cedric A. Collingwood Collection.

The holotype specimen of the new species *S. elhawagryi* is deposited at KSMA and a single paratype specimen is deposited at all the later museums except TAUI. An official permission from KSMA and WMLC has been obtained to examine some type materials according to their loan policy.

### Illustrations

Specimens were photographed by Erin Prado using a JVC KY-F70B 3CCD digital camera attached to a Leica M420 stereomicroscope. All digital images were processed using Auto-Montage (Syncroscopy, Division of Synoptics Ltd, USA) software. Images of the specimens are available in full color on www.antweb.org

No specific permits were required for the described field studies or for the surveyed locations which are not privately-owned or protected in any way or do not have endangered or protected species. *Solenopsis* specimens were collected by sifting tray and aspirator in Elqamh Park, Al Baha Province, southwestern Saudi Arabia. *Solenopsis saudiensis* and *S. sumara* specimens were examined in collections at King Saud Museum of Arthropods (KSMA) and World Museum Liverpool (WMLC). Official permission from KSMA and WMLC was obtained to examine type materials according to museum loan policy.

### Nomenclatural acts

The electronic edition of this article conforms to the requirements of the amended International Code of Zoological Nomenclature, and hence the new names contained herein are available under that Code from the electronic edition of this article. This published work and the nomenclatural acts it contains have been registered in ZooBank, the online registration system for the ICZN. The ZooBank LSIDs (Life Science Identifiers) can be resolved and the associated information viewed through any standard web browser by appending the LSID to the prefix “http://zoobank.org/”. The LSID for this publication is: urn:lsid:zoobank.org:pub: [D3189D0E-ECAA-44E9-9CCE-9E2E7748AC42]. The electronic edition of this work was published in a journal with an ISSN, and has been archived and is available from the following digital repositories: PubMed Central, LOCKSS.

## Results

### *Solenopsis elhawagryi* Sharaf & Aldawood, sp. n

urn:lsid:zoobank.org:act:36CC0F66-C76D-4E8C-A5B0-A56CD27761A2

#### Holotype worker (Major)

TL 2.37; HL 0.65; HW 0.52; SL 0.37; EL 0.05; PRW 0.30; ML 0.62; PL 0.17; PW 0.17; PPL 0.12; PPW 0.17; Indices: SI 71; CI 80

#### Paratype small workers

TL 1.55–1.82; HL 0.42–0.47; HW 0.35–0.50; SL 0.22–0.30; EL 0.02; PRW 0.17–0.25; ML 0.42–0.50; PL 0.12–0.17; PW 0.12–0.15; PPL 0.10–0.12; PPW 0.12–0.15; Indices: SI 50–88; CI 74–111 (16 measured).

#### Paratype major workers

TL 1.87–2.62; HL 0.50–0.65; HW 0.40–0.50; SL 0.30–0.40; EL 0.05; PRW 0.25–0.32; ML 0.50–0.77; PL 0.15–0.22; PW 0.12–0.20; PPL 0.10–0.15; PPW 0.12–0.20; Indices: SI 60–80; CI 76–88 (12 measured).

#### Paratype queen

TL 4.30; HL 0.75; HW 0.65; SL 0.47; EL 0.22; PRW 0.72; ML 1.40; PL 0.35; PW 0.27; PPL 0.25; PPW 0.30; Indices: SI 72; CI 87.

#### Type location

SAUDI ARABIA, Al Baha Province, Beljorashi Governorate, Elqamh Park, 19.91306°N, 41.90500°E, 1931 m.a.s.l. 17.v.2010 *(M. R. Sharaf leg.)*; King Saud Museum of Arthropods (KSMA), College of Food and Agriculture Sciences, King Saud University, Riyadh, Kingdom of Saudi Arabia.

#### Paratypes

20 workers, same locality and data as holotype, 1 (**MHNG** ); 1 ( **NHMB** ); 1 (**CASC)**; 1 (**WMLC)**; 1 (**BMNH**) and 15 (**KSMA**). In addition, 35 paratype specimens preserved in alcohol, 8 major workers and 27 minors (**KSMA**).

#### Diagnosis

Major workers can be easily separated from other Arabian *Solenopsis* by the color, head partially brownish yellow with the area in front of eyes yellowish and few scattered yellowish spots; posterior margin of first gastral tergite brownish. In addition, *elhawagryi* is similar to *omana*, both having the postpetiole with anteroventral projection, but the former species can be readily recognized from the later by the number of ommatidia of eyes which is four-five whereas in *omana*, eyes have seven ommatidia.

### Description

#### Major worker (Figs. 1–9)

**Figure 1:**
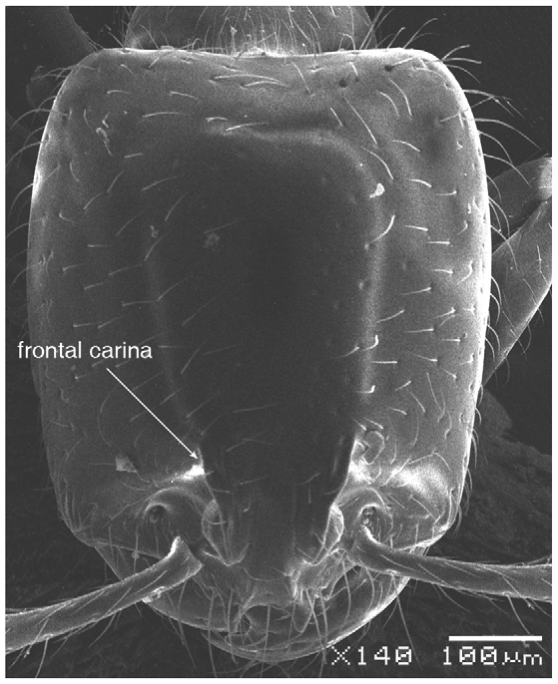
SEM of *Solenopsis elhawagryi* sp. n., major worker, head in full-face view.

**Figure 2:**
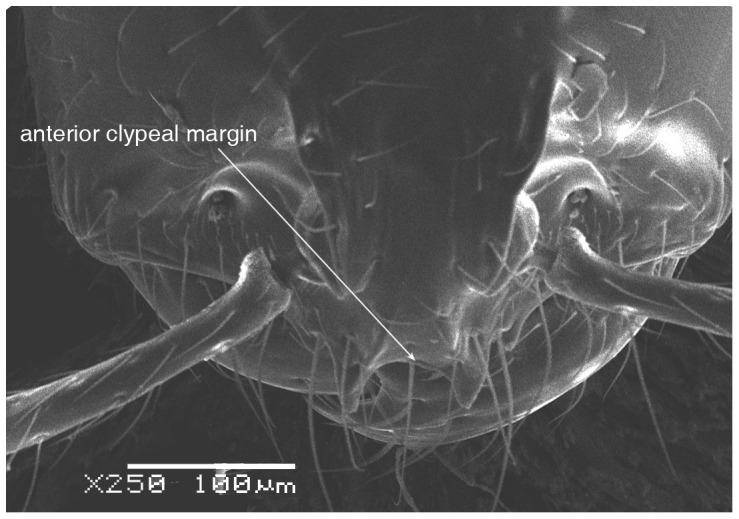
SEM of *Solenopsis elhawagryi* sp. n. major worker, clypeus.

**Figure 3:**
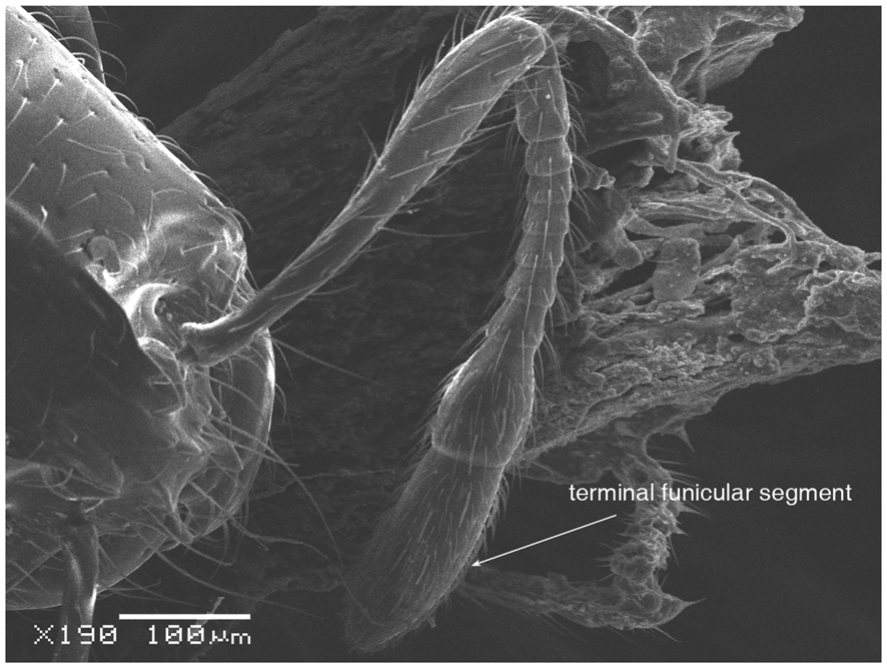
SEM of *Solenopsis elhawagryi* sp. n. major worker, antenna.

**Figure 4:**
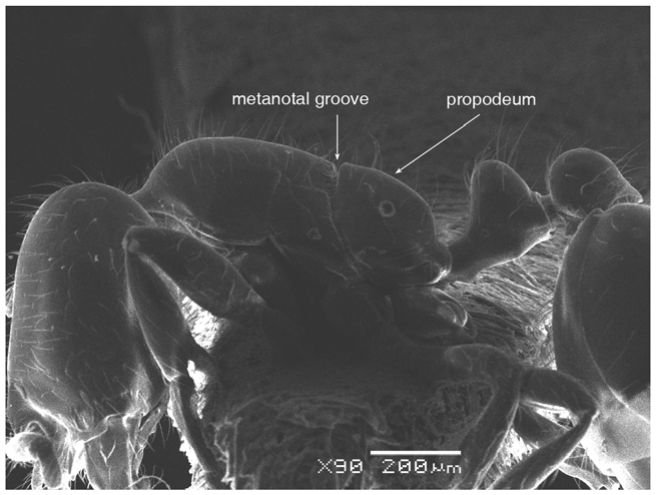
SEM of *Solenopsis elhawagryi* sp. n. major worker, body in profile.

**Figure 5:**
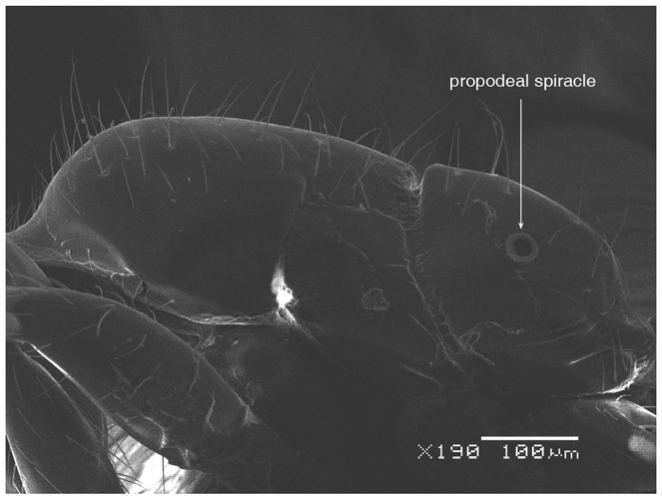
SEM of *Solenopsis elhawagryi* sp. n. major worker, mesosoma in profile.

**Figure 6:**
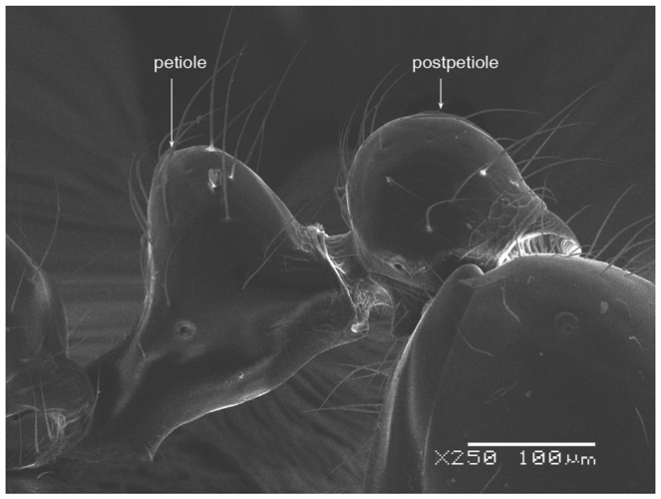
SEM of *Solenopsis elhawagryi* sp. n. major worker, petiole and postpetiole in profile.

**Figure 7:**
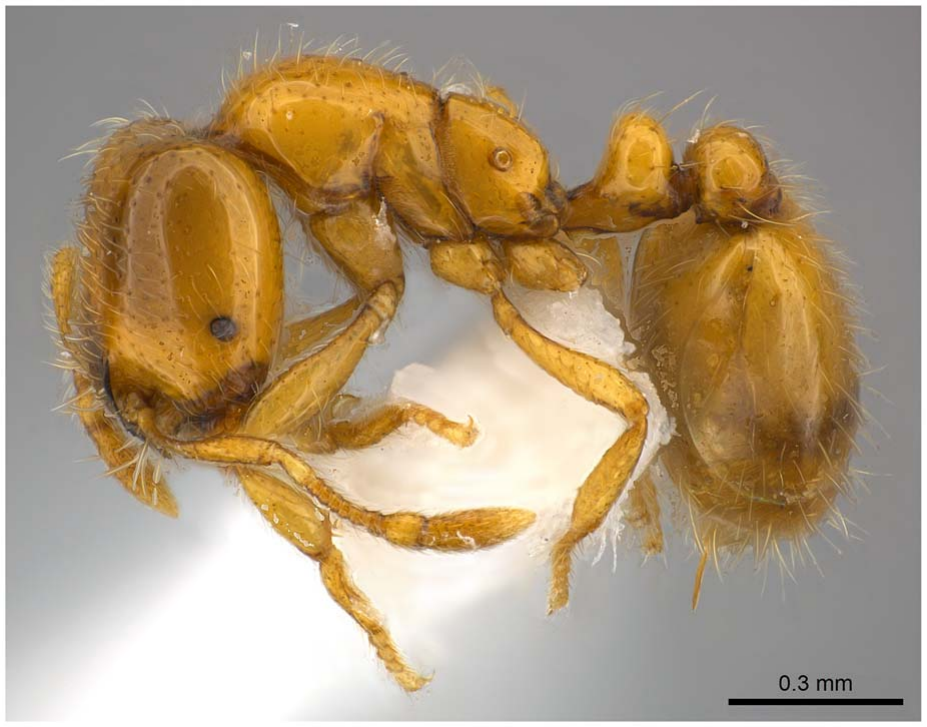
Automontage of *Solenopsis elhawagryi* sp. n., major worker, body in profile.

**Figure 8:**
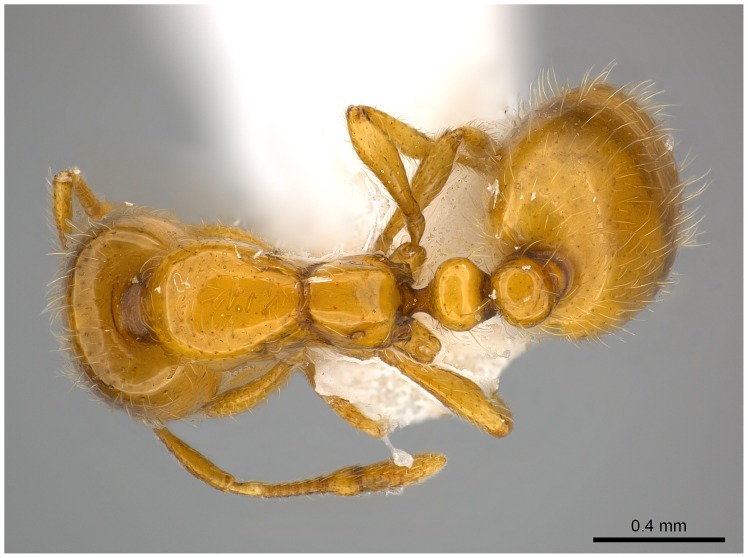
Automontage of *Solenopsis elhawagryi* sp. n., major worker, body in dorsal view.

**Figure 9:**
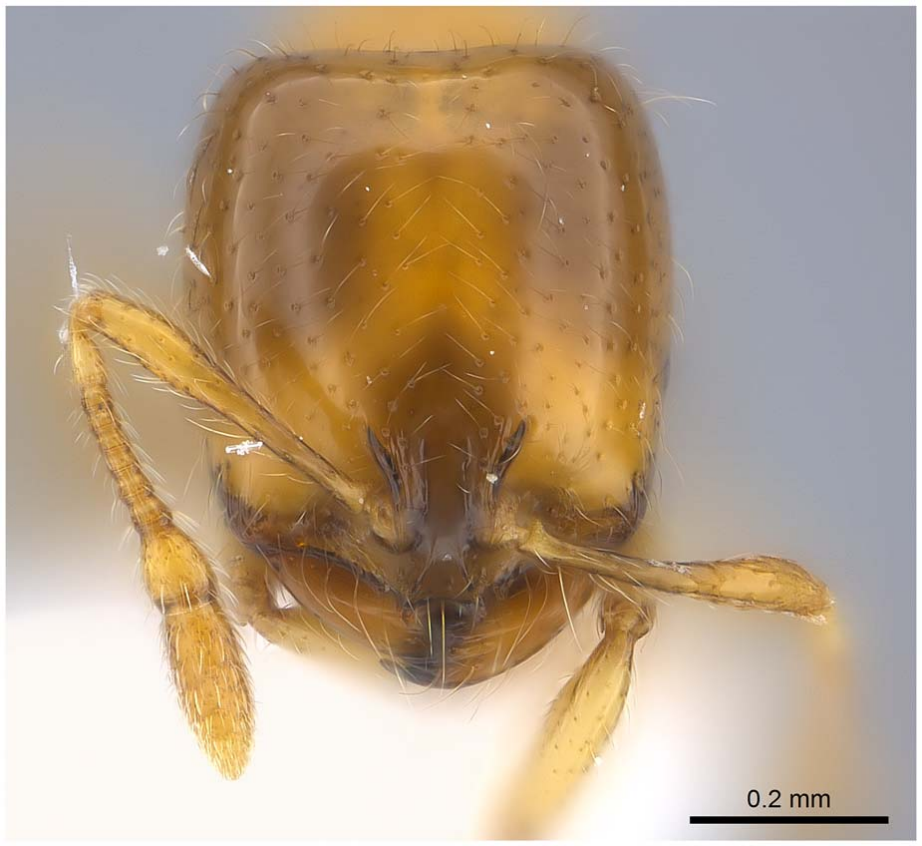
Automontage of *Solenopsis elhawagryi* sp. n., major worker, head in full-face view.

Head clearly longer than broad with weakly convex sides and nearly straight or very feebly concave posterior margin; cephalic dorsum smooth and shining with abundant short pitted setae; eyes small and oval, with four-five ommatidia; anterior clypeal margin with a central pair of stout projecting teeth and a lateral pair of short, broad basal blunt teeth; mandibles smooth and shiny, armed with four teeth, apical tooth largest followed by two subequal teeth, more widely separated, basal tooth smallest; antennae with terminal funicular segment long and about 3 times longer than proceeding segment; frontal carina much reduced. Metanotal groove a V-shape, with a narrow, acute cleft; propodeum outline a continuous curve in lateral view; propodeal spiracle circular. Petiolar peduncle with a small ventral tooth, high node and rounded dorsum, in dorsal view and about twice as broad as long. Postpetiole profile high and slightly spherical, with a distinct anteroventral tooth-like process bearing few setae; dorsum with a weak, superficial alveolate pattern; pilosity of mesosoma, petiole, postpetiole and gaster relatively abundant and long. Coloration overall yellow; head partially brownish yellow with the area in front of eyes yellowish and few scattered yellowish spots; posterior margin of first gastral tergite brownish. Overall smooth and shining.

#### Minor worker (Figs. 10–18)

**Figure 10:**
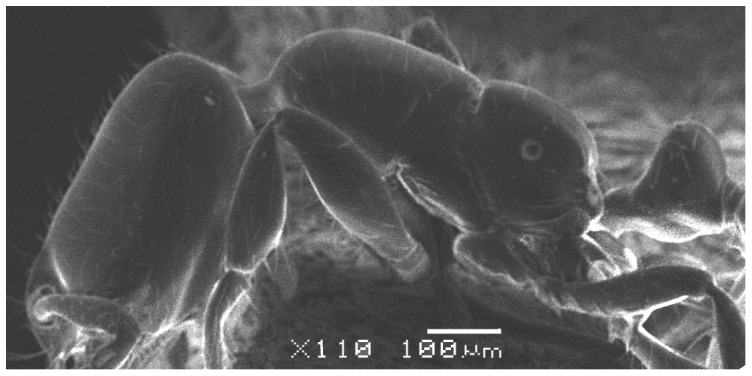
SEM of *Solenopsis elhawagryi* sp. n. minor worker, body in profile.

**Figure 11:**
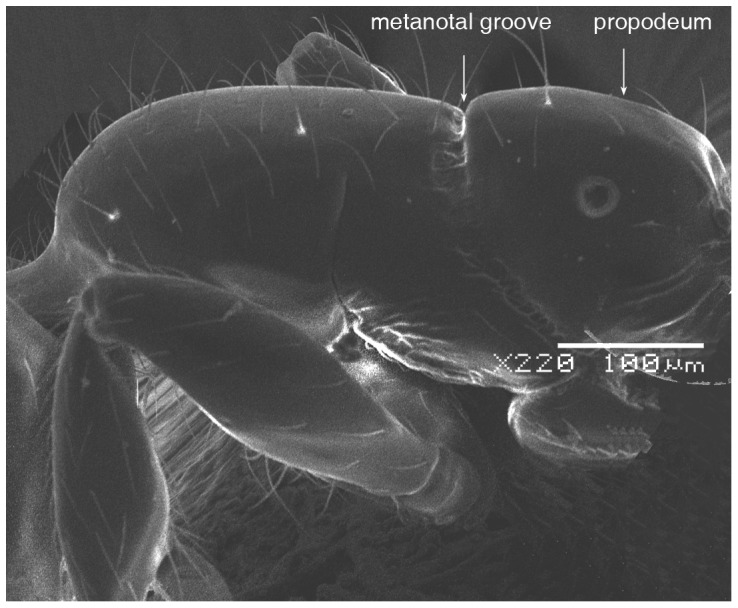
SEM of *Solenopsis elhawagryi* sp. n. minor worker, mesosoma in profile.

**Figure 12:**
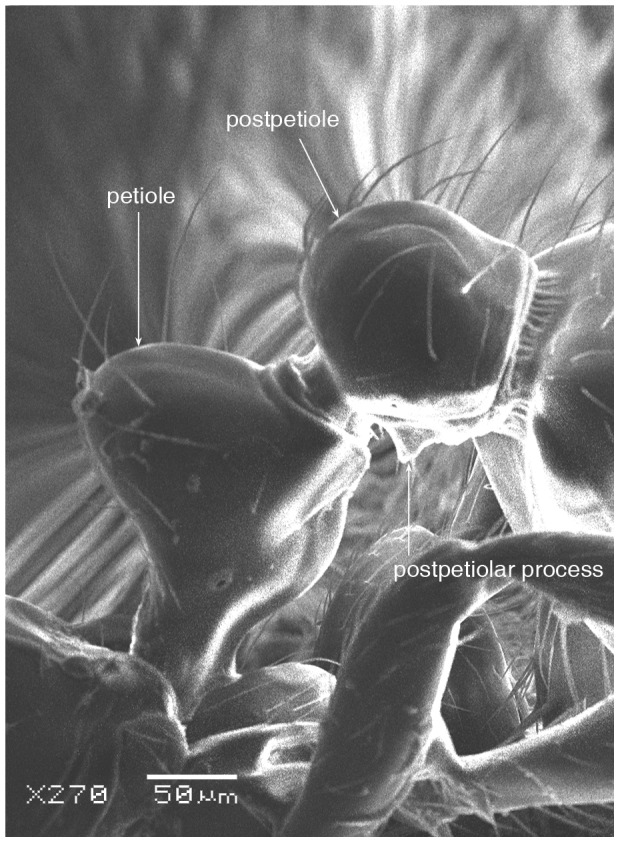
SEM of *Solenopsis elhawagryi* sp. n. minor worker, petiole and postpetiole in profile.

**Figure 13:**
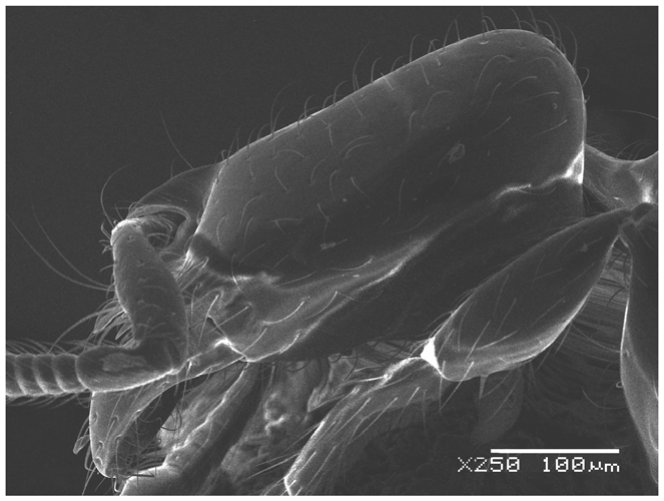
SEM of *Solenopsis elhawagryi* sp. n. minor worker, head in profile.

**Figure 14:**
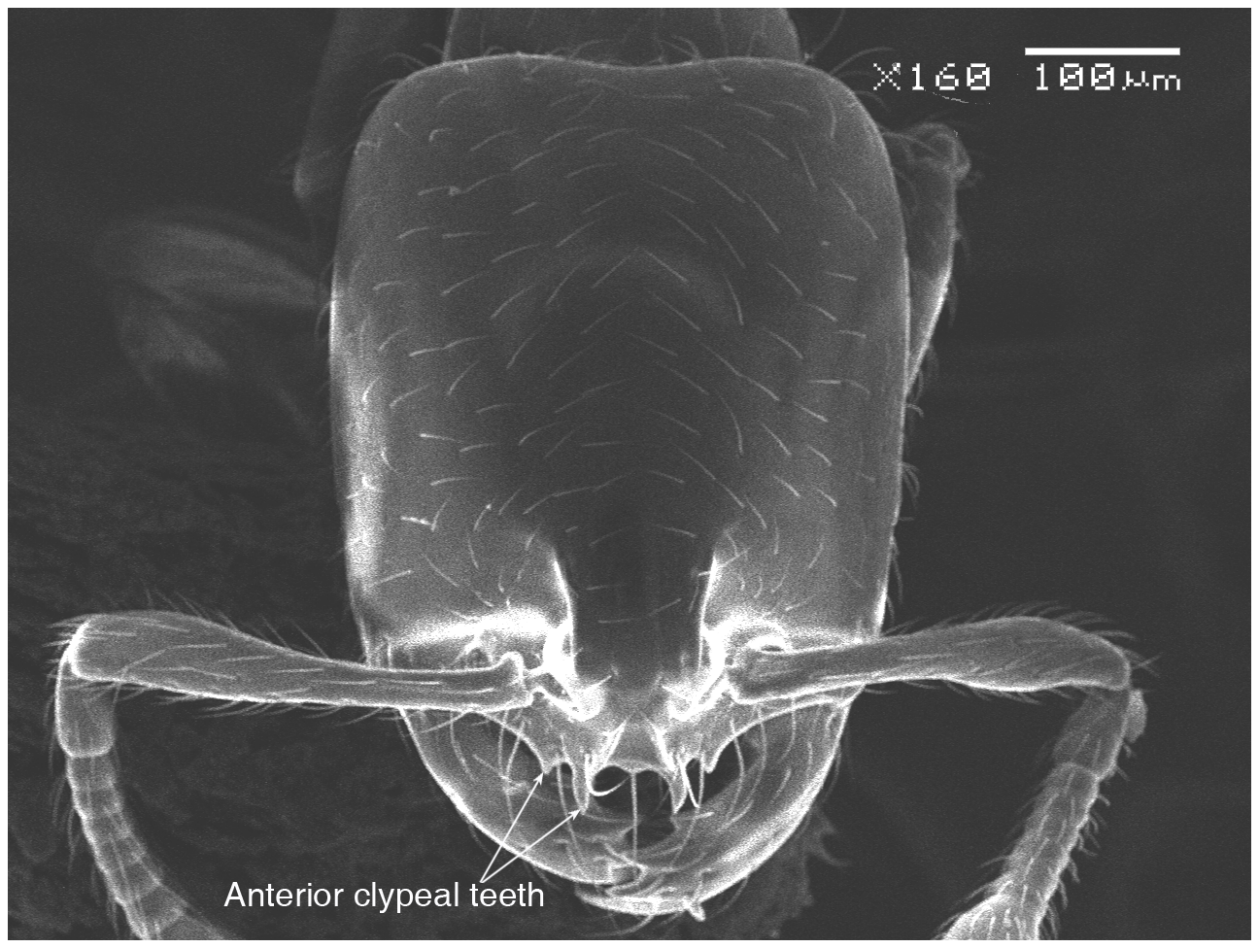
SEM of *Solenopsis elhawagryi* sp. n. minor worker, head in full-face view.

**Figure 15:**
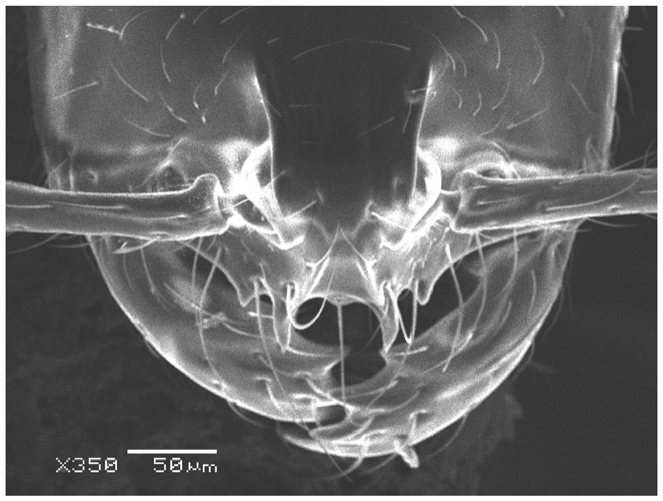
SEM of *Solenopsis elhawagryi* sp. n. minor worker, clypeus.

**Figure 16:**
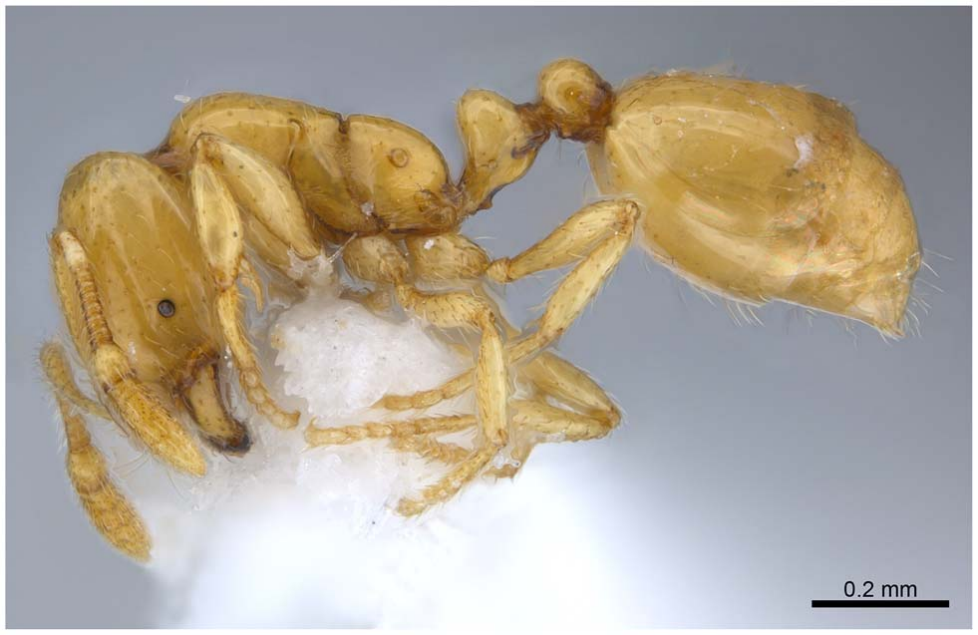
Automontage of *Solenopsis elhawagryi* sp. n., minor worker, body in profile.

**Figure 17:**
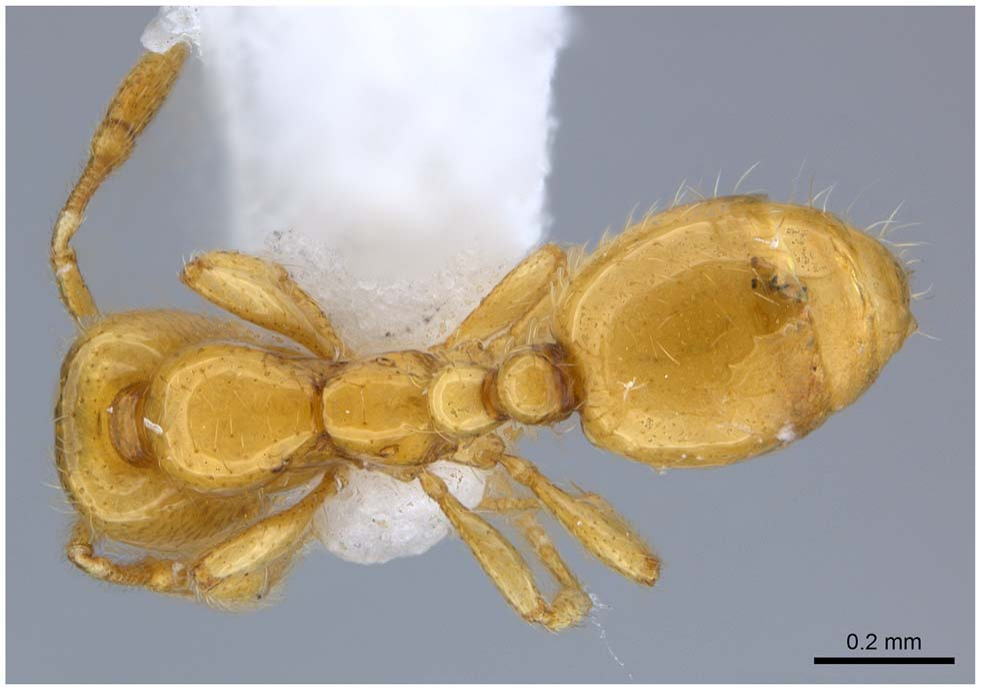
Automontage of *Solenopsis elhawagryi* sp. n., minor worker, body in dorsal view.

**Figure 18:**
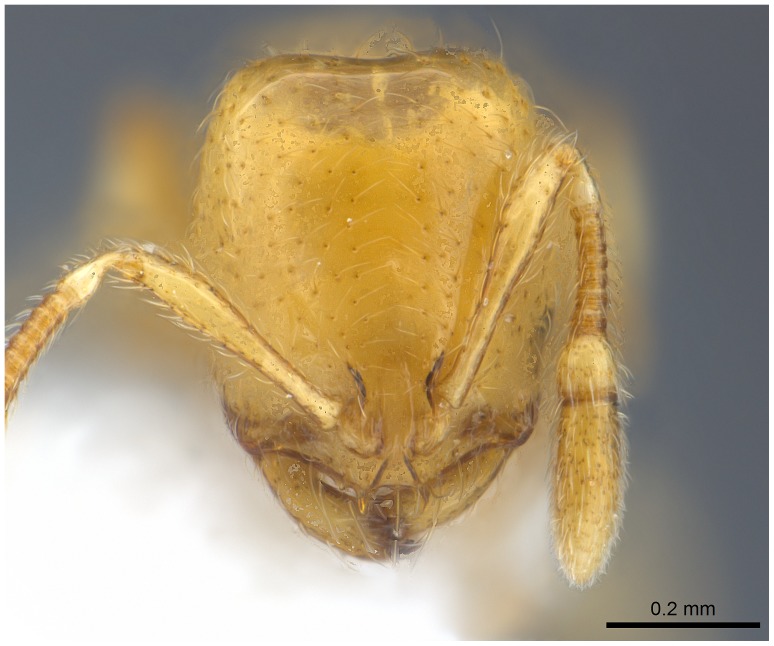
Automontage of *Solenopsis elhawagryi* sp. n., minor worker, head in full-face view.

Most characters are as in the major workers but: head little longer than broad, with feebly convex sides; eyes proportionally smaller, with four ommatidia; central clypeal teeth widely separated, long, and acute; body pilosity shorter, especially on the head.

#### Queen (alate) (Figs. 19–21)

**Figure 19:**
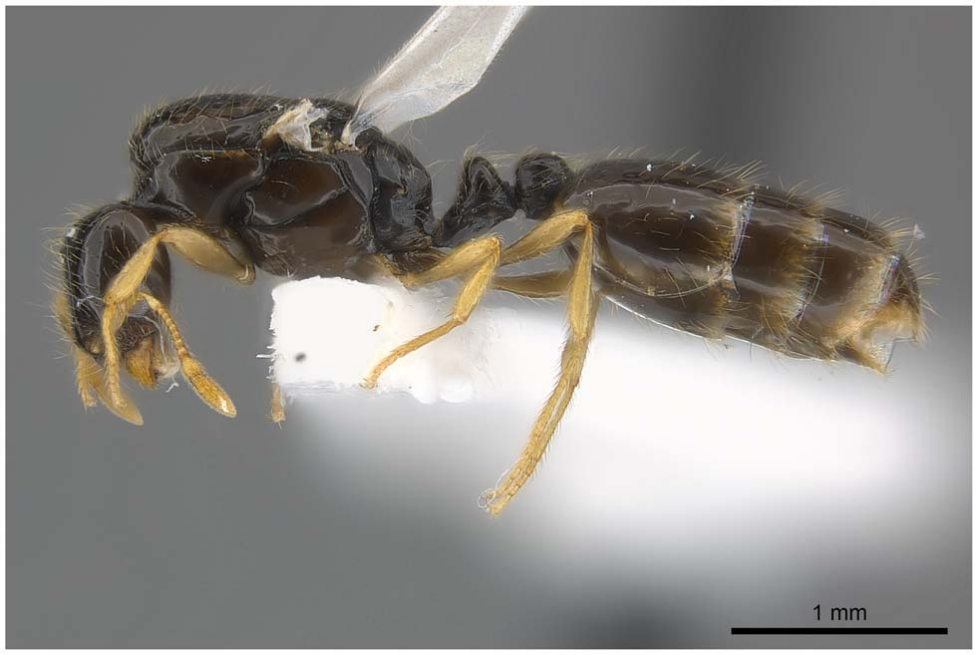
Automontage of *Solenopsis elhawagryi* sp. n., queen, body in profile.

**Figure 20:**
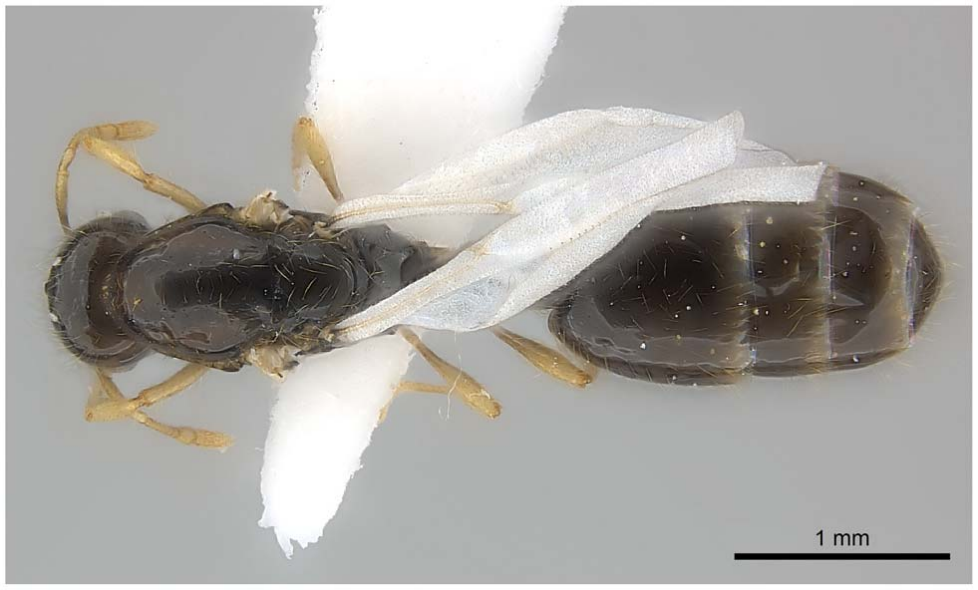
Automontage of *Solenopsis elhawagryi* sp. n., queen, body in dorsal view.

**Figure 21:**
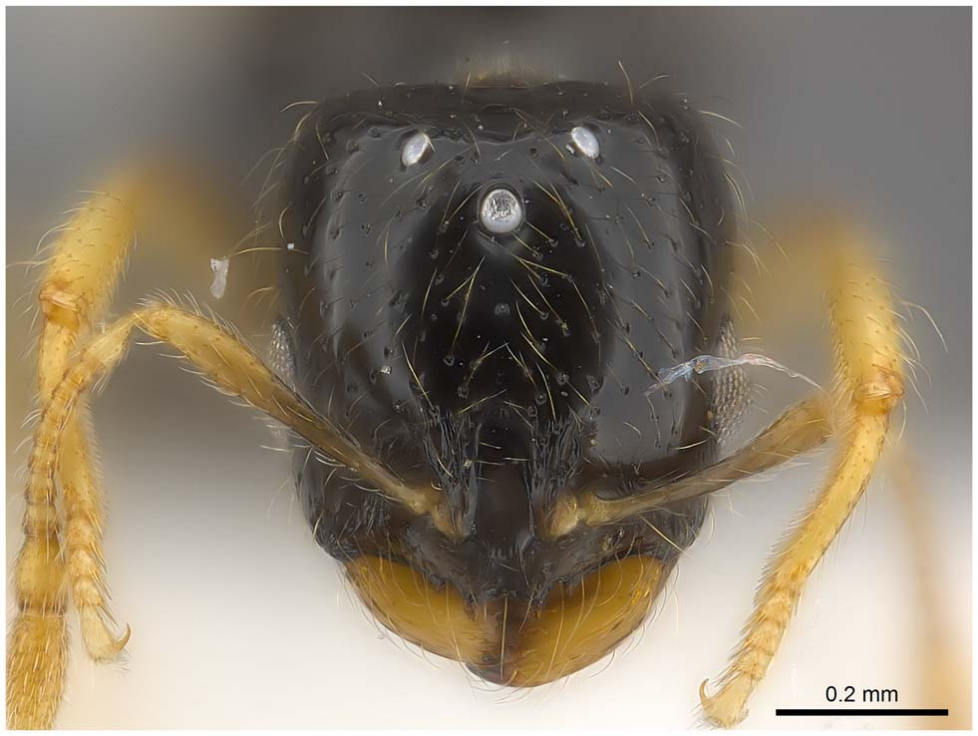
Automontage of *Solenopsis elhawagryi* sp. n., queen, head in full-face view.

Head distinctly longer than broad with slightly convex sides and a feebly concave posterior margin; cephalic dorsum smooth and brilliant with abundant inwardly-directed setae, central setae much longer; the three ocelli large and whitish; eyes very large (about 0.33× HW) with more than 25 ommatidia in the longest row; anterior clypeal margin with a central pair of stout projecting teeth, and a lateral pair of short, broad basal blunt teeth; mandibles faintly longitudinally striated, armed with four teeth, the distal one being the largest, and the second and third being subequal and slightly far apart; basal tooth the smallest; antennal scape short, just surpassing the level of posterior margin of eyes; frontal carinae much reduced. Mesosoma robust with abundant, short, suberect setae. Petiolar dorsum narrow and convex in profile. Postpetiolar dorsum wide and convex. Petiole and postpetiole have abundant, long, backwardly-directed setae and petiole with a small distinct anteroventral toothlike process which bears few long setae; both smooth and shining, with imbricate sculpture on sides. Gaster long and robust. Color uniformly dark brown, funiculus, distal half of scape, legs and mandibles yellowish, basal half of scape brownish yellow, mesopleura light brownish.

#### Affinities

*Solenopsis elhawagryi* appears closest to *S. omana* from Oman and UAE and *S. dentata* from Israel. All three species share the following characters: anterior clypeal margin with a central pair of stout projecting teeth and a lateral pair of short, broad basal blunt teeth; head longer than broad; petiole node high and rounded in profile, peduncle with a small ventral tooth, node from above distinctly broader than long; postpetiole in profile with a characteristic tooth-like anteroventral flange, with some projecting setae.

**Figure 22:**
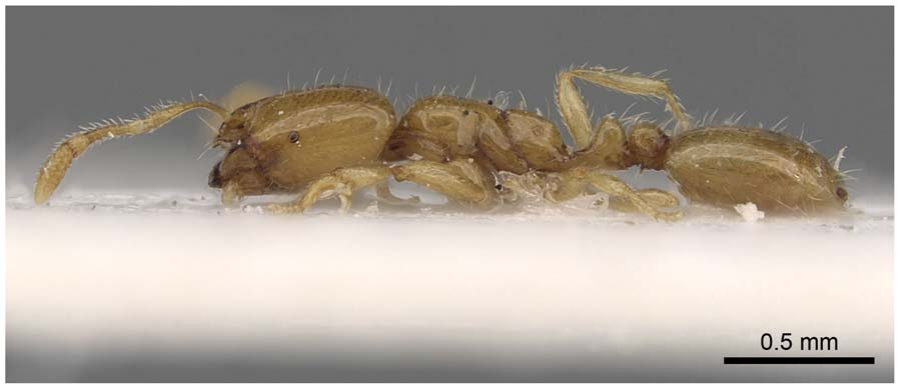
Automontage of *Solenopsis dentata*, worker, body in profile.

*Solenopsis elhawagryi* can be easily distinguished from *S. dentata* (type examined, **Figs. 22** **–** **24**) by the following characters: the postpetiolar tooth-like anteroventral flange is simple but distinct, whereas in *dentata* it is more acute, longer and clearly curved, and very characteristic; the eyes have four-five ommatidia while *dentata* has 7 ommatidia; the propodeal outline is a continuous curve in lateral view, whereas in *dentata* the propodeal dorsum meeting the declicity in an obtuse angle; *elhawagryi* has a characteristic V-shaped, narrow and acute metanotal groove, while the metanotal groove in *dentata* is less acute. In addition, *elhawagryi* has less abundant body pilosity while *dentata* has more abundant pilosity. Moreover, *elhawagryi* has a lower scape index, SI 60–80 against SI 81–89; the cephalic index is slightly lower, CI 76–88 against CI 82–89.

**Figure 23:**
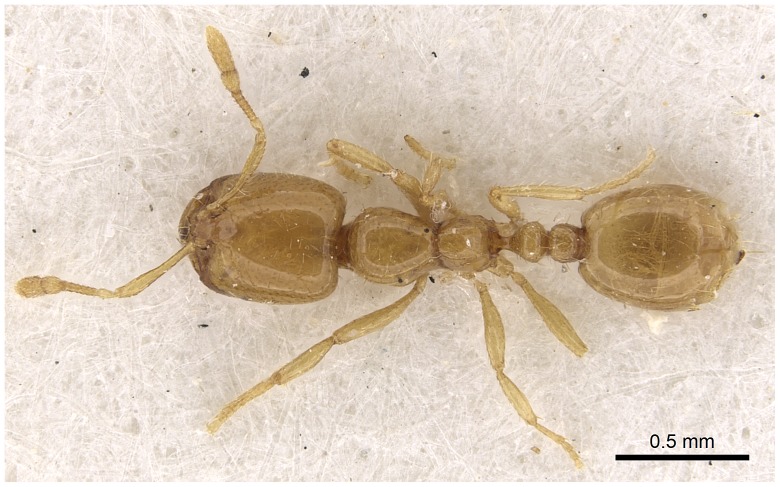
Automontage of *Solenopsis dentata*, worker, body in dorsal view.

**Figure 24:**
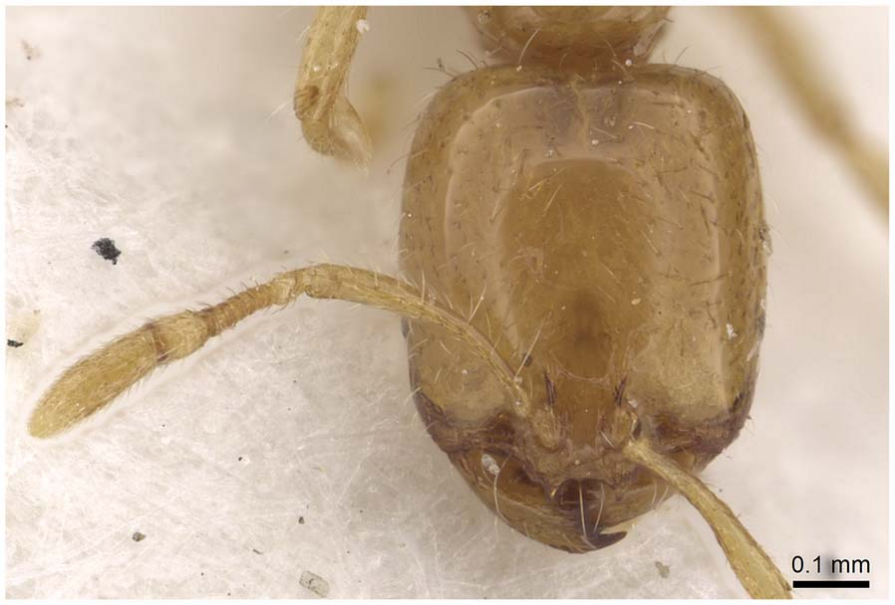
Automontage of *Solenopsis dentata*, worker, head in full-face view.

Comparing the queen of *elhawagryi* and *dentata*, both are similar in color (dark brown or blackish brown) and body measurements, but consistent differences occur. The eyes of *elhawagryi* are distinctly larger (about 0.33× HW), with more than 25 ommatidia in the longest row, the *dentata* eyes are about 0.25× HW, with 18 ommatidia in the longest row; *elhawagryi* has a lower cephalic index, CI 87 versus CI 93–95, and a slightly higher scape index SI 72 versus SI 67–69.

Comparing *elhawagryi* with *omana*, the major worker eyes have four-five ommatidia, compared with seven ommatidia; *elhawagryi* has the head in profile appearing thick with a flat dorsal surface, and a distinctly convex ventral surface, while in *omana* the head in profile appears narrow with a flat ventral surface; the cephalic index tends to be larger, CI 76–88 versus CI 79–81.

#### Etymology

This new species is named in the honor of Prof. Magdi El-Hawagry (Cairo University and Al Baha University).

#### Habitat and Biology

The type locality is a forest called Elqamh Park, Beljorashi Governorate, Al Baha Province, Kingdom of Saudi Arabia, with many water pools, and soil has significant degree of humidity after heavy rainy season. The area has much native vegetation including: *Acacia origena* A. Hunde, *Acacia negrri* Pichi-Sermoli, *Solanum* sp., and *Juniperus procera* Hochst. Ex Endle. The type specimen was taken from a nest under a stone on loose soil next to a large and old *Acacia* tree, the nest contained tens of major and minor workers, and two alate queens. No additional nests were found despite extensive surveys in the area. In addition, we were unable to collect foraging workers near the nest. Several other ant species were found in the same habitat including: *Crematogaster affabilis* Forel, *Monomorium mayri* Forel, *M. salomonis* (L.), *M. monomorium* group, *Tetramorium sericeiventre* Emery, and *Lepisiota obtusa* Emery.

Although this new species has not been found in any area other than the type locality and despite intensive collecting efforts in the surrounding areas in Asir Mountains, it is not certain that this species is endemic for the region, but it is hoped that future collecting attempts in adjacent areas and countries can give a true idea of species status and distribution.

### *Solenopsis geminata* (Fabricius, 1804) (Figs. 25–27)

**Figure 25:**
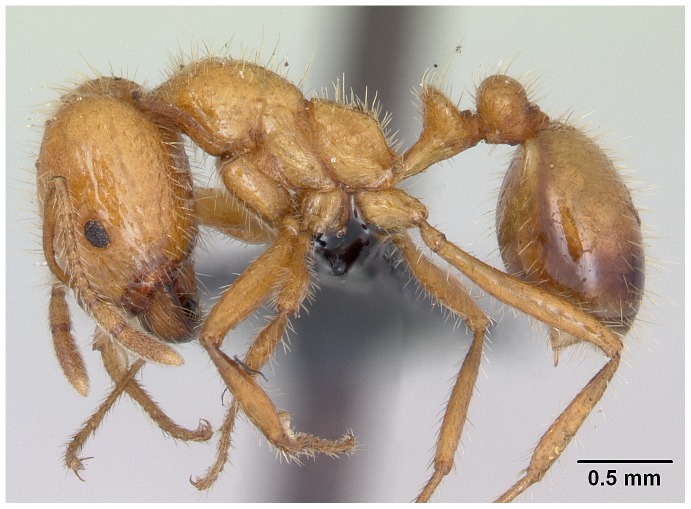
Automontage of *Solenopsis geminata*, major worker, body in profile.

**Figure 26:**
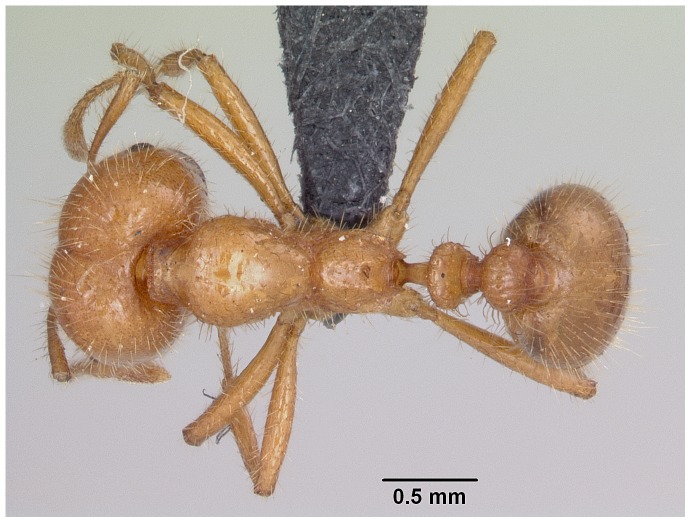
Automontage of *Solenopsis geminata*, major worker, body in dorsal view.

**Figure 27:**
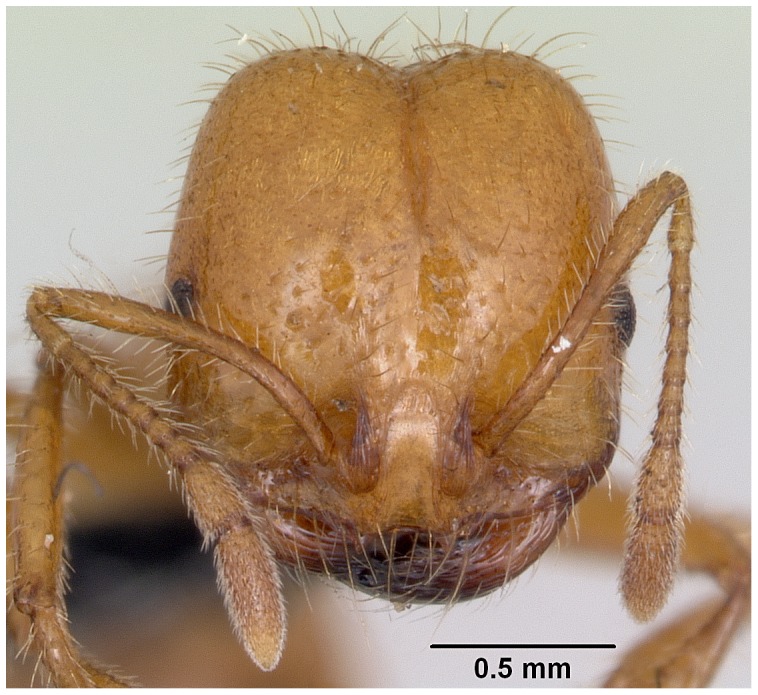
Automontage of *Solenopsis geminata*, major worker, head in full-face view.

*Atta geminata* Fabricius, 1804: 423. Queen, CENTRAL AMERICA [Not examined]

*Solenopsis geminata* Mayr, 1863:453 [15].

#### Diagnosis

*Solenopsis geminata* can be distinguished from other Arabian species by the combination of the following characters: head subquadrate to subtrapezoidal; posterior margin of head with deep angular median emargination; mandibles thick and strongly curved mesad, especially in largest individuals; propodeal dorsum with distinct dorsolateral carinae; petiolar ventral process small.

#### Workers

Polymorphic; head subquadrate to subtrapezoidal, with straight to feebly convex sides and parallel to feebly divergent anteriad, often slightly indented just anterior to eyes; posterior margin of head with deep angular median emargination between nearly hemispherical lobes; eyes small relative to head, with 9–11 ommatidia in longest row; median clypeal tooth rudimentary; carinal teeth thick at base, strongly protruding; mandibles thick and strongly curved mesad, especially in largest individuals. Pronotal profile strongly convex and at most weakly angular; promesonotal suture distinct, approximately right-angular to weakly obtuse-angular; metanotal groove impressed; propodeal profile nearly diamond-shaped, with flat to weakly concave dorsum; propodeal dorsum of large and smaller workers with distinct dorsolateral carinae. . Cephalic and promesonotal pilosity abundant. Color very variable, from concolorous orange-red with only posterior margin of gaster dark brown, to nearly concolorous brownish black with only head near base of mandibles and appendages reddish-brown. Smaller workers darker and more uniformly colored than larger ones.

#### Queen

TL 7.5–8.0 mm [16] Head one-sixth broader than long, quadrate, little wider behind eyes than in front of them, sides very feebly convex from eyes to posterior corners of head, straight or nearly straight in front of eyes and meeting anterior border of head at sharp angle. posterior corners of head well-marked, posterior margin flat with narrow and shallow median impression, frontal furrow short, clearly marked only for about half distance from median ocellus to base of frontal lobes, thereafter becoming shallow and indistinct; ocelli large and prominent; clypeus feebly projecting, carinal teeth very stout, blunt, clypeal border between them with shallow concave impression; lateral denticles small, feebly defined; masticatory margin of mandibles with three large teeth and usually rudiment of fourth one; eyes large, strongly convex, irregularly oval in outline, posterior border reaching a point half way between posterior and anterior borders of head; antennal scape just reaches lateral ocellus; funicular segments and club as in major worker. Mesosoma robust, elliptical, maximum width three-fifths of its length, slightly narrower than head (eyes excluded); mesonotum in profile with straight posterior half and convex anterior portion overhangs pronotum; Scutellum as high as mesonotum, feebly convex with short, perpendicular posterior face; propodeal angle well-defined, obtuse, with propodeal dorsum and declivity of about equal length. Petiolar nodes similar to those of major worker except thicker peduncle, node of petiole slightly lower and postpetiole with obtuse, conical, ventral projection on sides. Seen from above, petiole and postpetiole strongly transverse and with equal width. Gaster as in major worker.

#### Male

TL 5.80 mm [16]. Head trapezoidal, maximum width (including eyes) approximately one-fourth greater than its length; eyes very large, strongly convex and oval in outline, occupying more than one-half head sides, their anterior border reaching mandibular insertions; ocelli very large and prominent, lateral ocelli with shallow concave impression between them; anterior clypeal margin nearly straight; mandibles small, linear, bidentate; antennal scape about one and one-half times as long as broad, cylindrical; first funicular segment sub globose, broader than scape; second funicular segment more than twice as long as broad, third joint one and one-half times as long as broad, remaining segments more than twice as long as broad. Mesosoma elliptical, its greatest width two-thirds of its length; anterior part of mesonotum in profile greatly swollen and overhangs pronotum; propodeum slightly rounded, basal face strongly convex transversely and slightly convex longitudinally, declivity flat, virtually perpendicular. Petiolar node in profile low but with acute summit, anterior face not sharply separated from thick peduncle, posterior face perpendicular; summit of node from posterior view with broad, shallow median impression. Postpetiole in profile as high as node of petiole, about one and one-half times as high as long with long, backward sloping anterior face; postpetiole nearly three times as broad as long. First gastral segment truncate at base but not impressed.

#### Distribution

A species endemic to Central American that has spread through commerce to many countries [17]–[19]. It was collected for the first time in Arabia from Dubai [11] where it was an irritating nuisance to racing horses and camels.

### *Solenopsis omana* Collingwood & Agosti, 1996 (Figs. 28–31)

**Figure 28:**
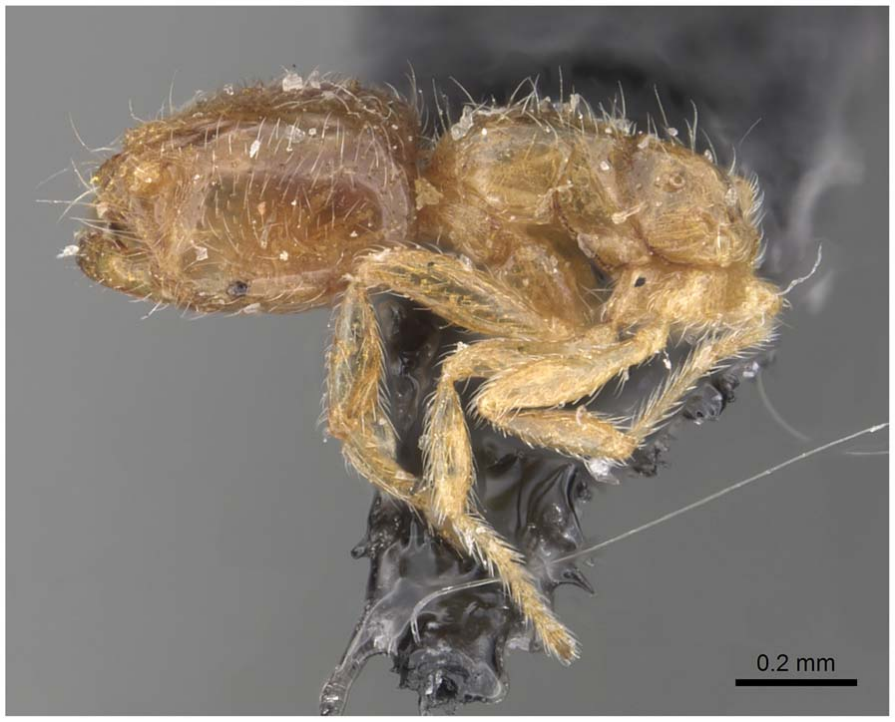
Automontage of *Solenopsis omana*, major worker, head and mesosoma in profile.

**Figure 29:**
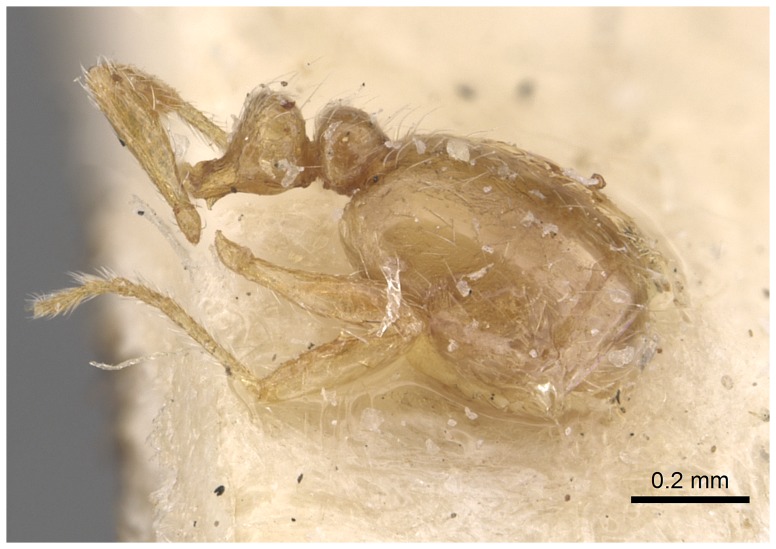
Automontage of *Solenopsis omana*, major worker, petiole, postpetiole and gaster in profile.

**Figure 30:**
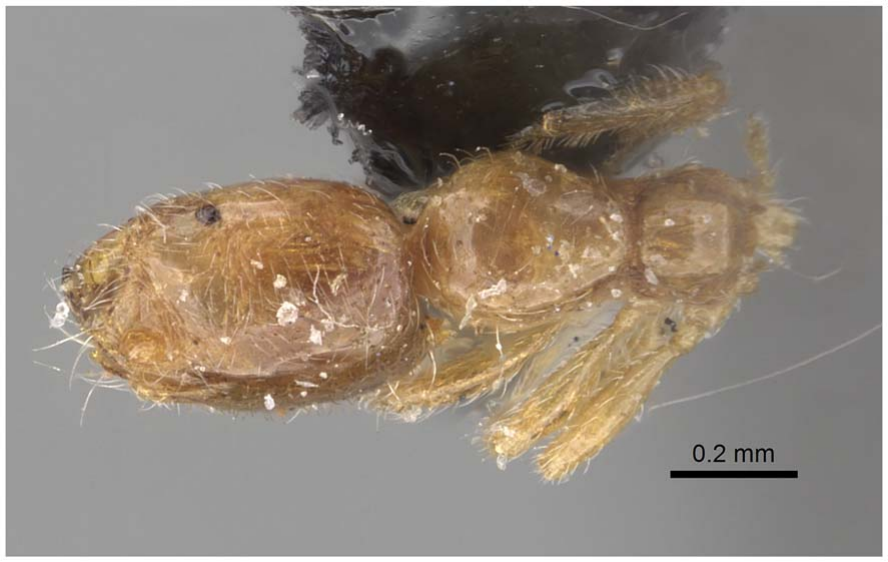
Automontage of *Solenopsis omana*, major worker, head and mesosoma in dorsal view.

**Figure 31:**
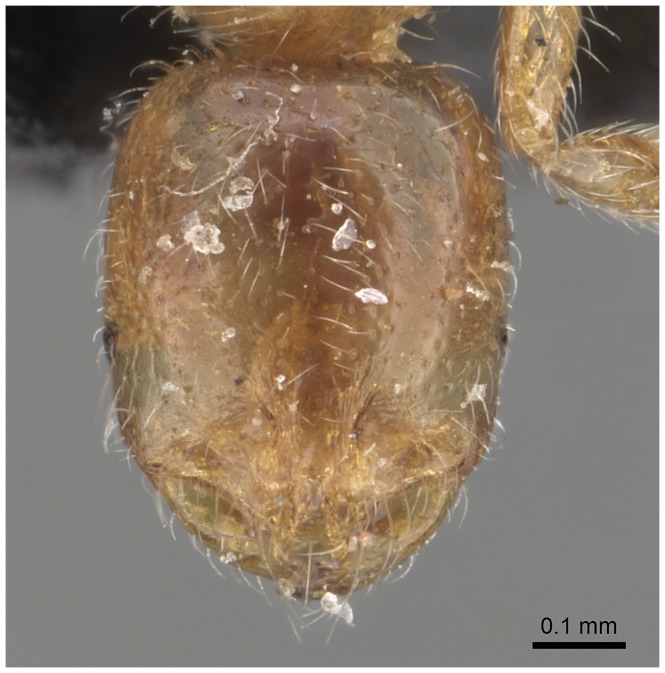
Automontage of *Solenopsis omana*, major worker, head in full-face view.

*Solenopsis omana* Collingwood & Agosti, 1996: 258. Holotype worker, OMAN, Ruwi, 15.II.1985, *(R. Braund)* (TAUI) [examined] Paratypes: OMAN: 5 workers, same series as holotype. – UNITED ARAB EMIRATES: 1 worker, Suneira, 25°12′N 55°33′E, 15.IY.1991, *(]. Gosse)* [not in WML, presumably lost].

#### Holotype major worker [10]

TL 2.0; HL 0.57; HW 0.44; SL 0.36; SI 82; CI 79–81.

#### Diagnosis

This species is closest related to *elhawagryi*, but is quickly separated by the eyes which having seven ommatidia whereas eyes of *elhawagryi* with four-five ommatidia.

#### Description of major worker

Head subrectangular in dorsal view, narrow with a flat ventral surface in profile, with nearly straight posterior margin and feebly convex sides; eyes with seven ommatidia; central clypeal teeth widely spaced, short and blunt; teeth at each side very small projections. Metanotal groove deep and making an acute angle in profile; propodeal dorsum about 1.5× longer than propodeal declivity. Petiolar node higher than postpetiolar node; postpetiole with an anteroventral small projecting teeth in profile; All body surfaces with long setae which appear as a fringe surrounding posterior margin of head and genae; head smooth and shining with scattered large punctures, rest of body faintly sculptured or unsculptured; color dirty yellow, head and first gastral tergite with yellow brownish tint.

#### Remarks

Collingwood and Agosti [10] did not mention whether the described caste was a major or minor worker, but the measurements and the short, blunt, widely separated central clypeal teeth are shared by major workers of its congener, *S. elhawagryi*. The color also was not stated although they compare it to the yellow *S. orbula*. That, however, has minute eyes and sharp clypeal teeth. The original paper (p. 301) stated all type material was deposited in the NHMB but no specimens denoted as types can be found there (Daniel Burkhardt, Isabelle Pfander & Guy Knight, pers. comm.). Additionally, no type material exists at WMLC. Finally, the holotype was found deposited in the Tel Aviv University entomological collection, Israel.

**Figure 32:**
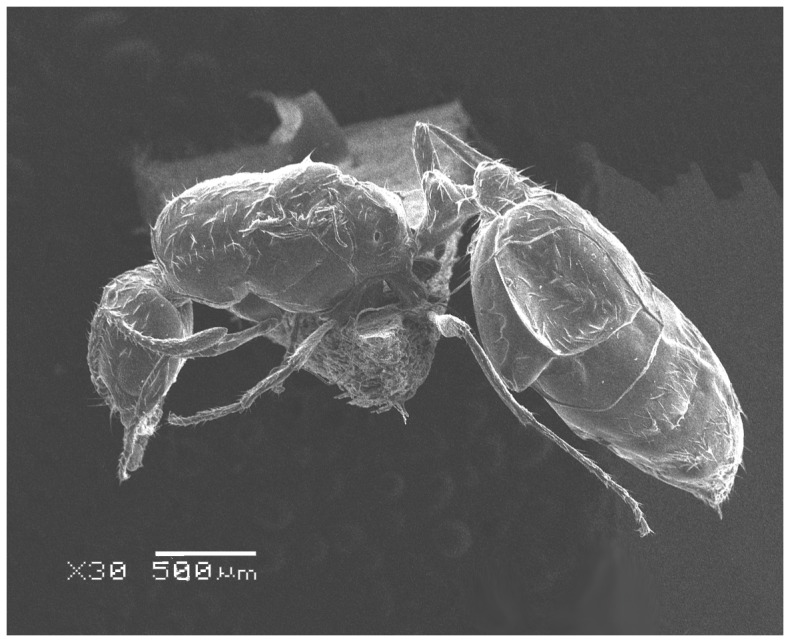
SEM of possibly queen of *Solenopsis omana*, body in profile.

A single dealated queen is in WMLC with an identification label written by C. A. Collingwood (pers. comm.) that denotes it as *S. omana* and has the locality information, Oman, Hayma Desert. This queen (**Figs. 32** **,** **33**) possesses the postpetiolar ventral teeth which support the hypothesis of being the queen of this ambiguous taxon. When *S. omana* is rediscovered and queens are collected in association with workers, then the real status of this single queen can be confirmed.

**Figure 33:**
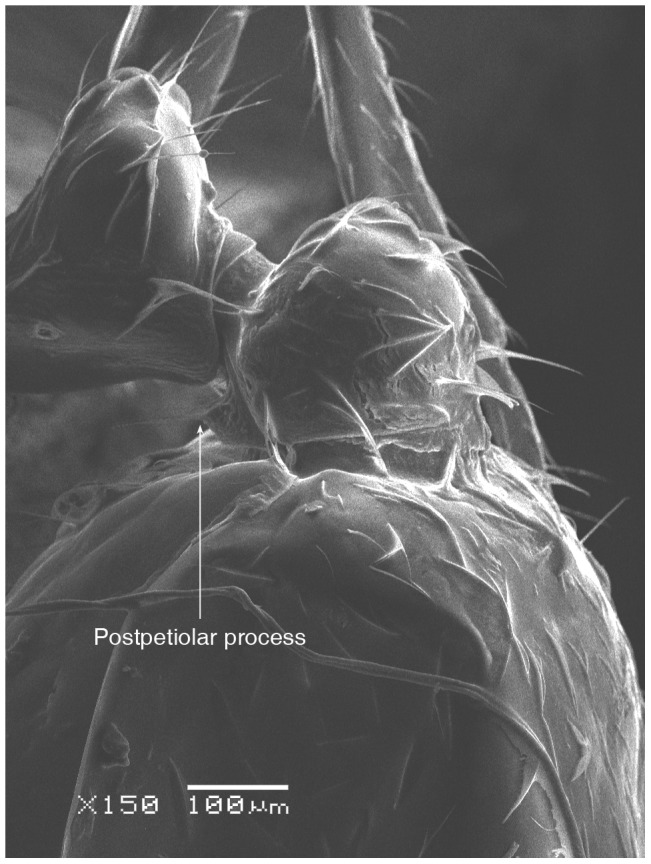
SEM of possibly queen of *Solenopsis omana*, postpetiole in profile.

#### Description

TL 4.50; HL 0.75; HW 0.65; SL 0.42; EL 0.20; SI 65; CI 87; ML 1.25; PL 0.22; PW 0.25; PPL 0.20; PPW 0.27

#### Dealated queen

Head distinctly longer than broad with concave posterior margin and convex sides; eyes large (EL 0.20), about one third of the head length, with 16 ommatidia in the longest row; anterior clypeal margin with a central pair of stout projecting teeth, and a pair of short, broad basal blunt teeth; petiole in profile pedunculate with a narrow and nearly pointed node; postpetiolar node lower than petiolar node and with a distinct small ventral tooth provided with setae; coloration brown, mandibles and legs yellowish.

### *Solenopsis saudiensis* Sharaf & Aldawood 2011 ( Figs. 34–42)

**Figure 34:**
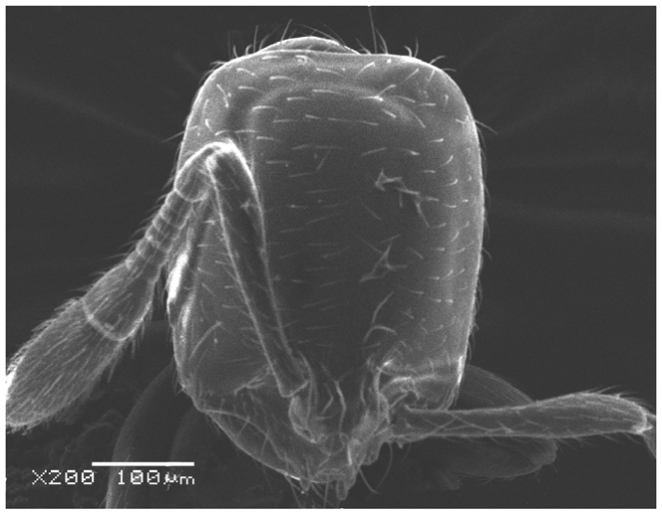
SEM of *Solenopsis saudiensis*, worker, head in full-face view.

**Figure 35:**
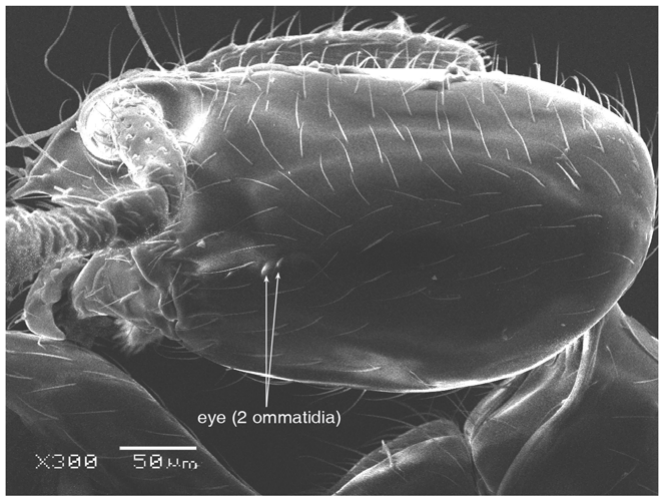
SEM of *Solenopsis saudiensis*, worker, head in profile.

**Figure 36:**
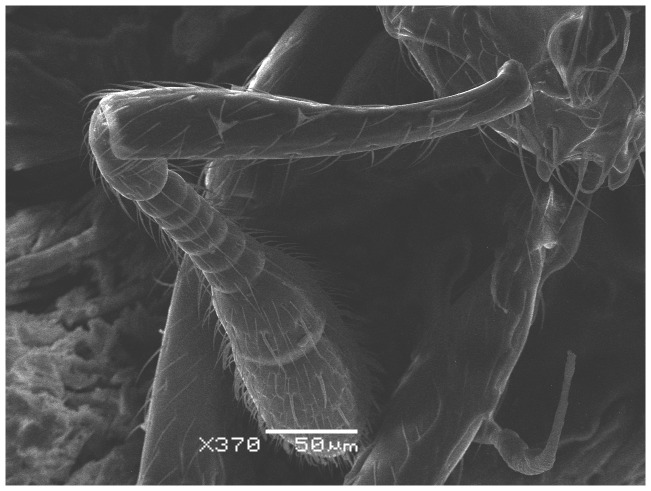
SEM of *Solenopsis saudiensis*, worker, antenna.

**Figure 37:**
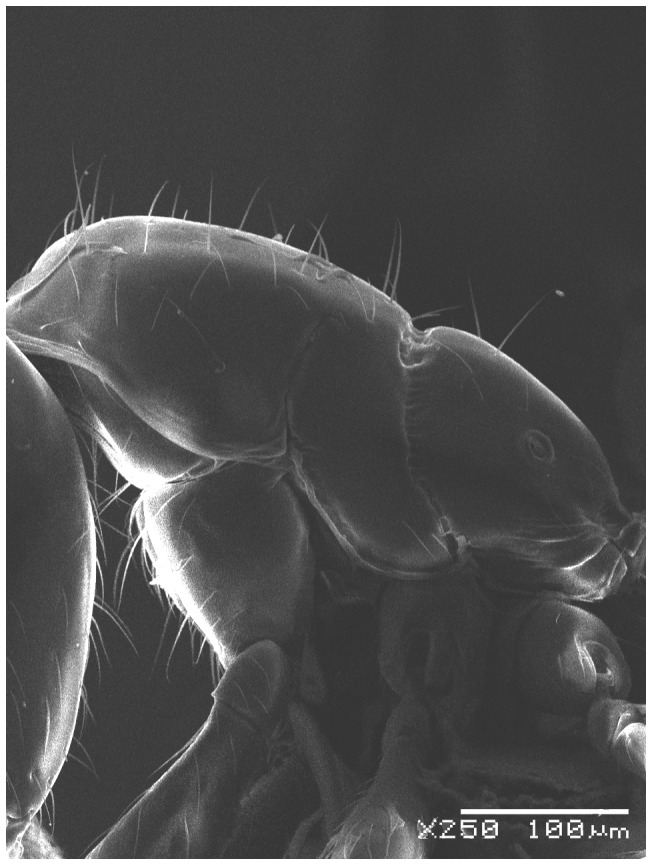
SEM of *Solenopsis saudiensis*, worker, mesosoma in profile.

**Figure 38:**
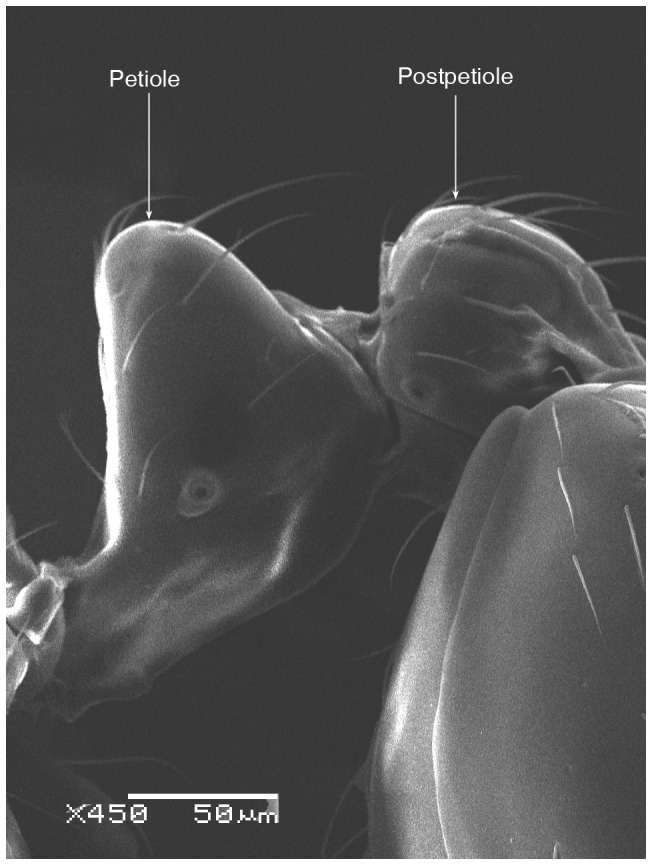
SEM of *Solenopsis saudiensis*, worker, petiole and postpetiole in profile.

**Figure 39:**
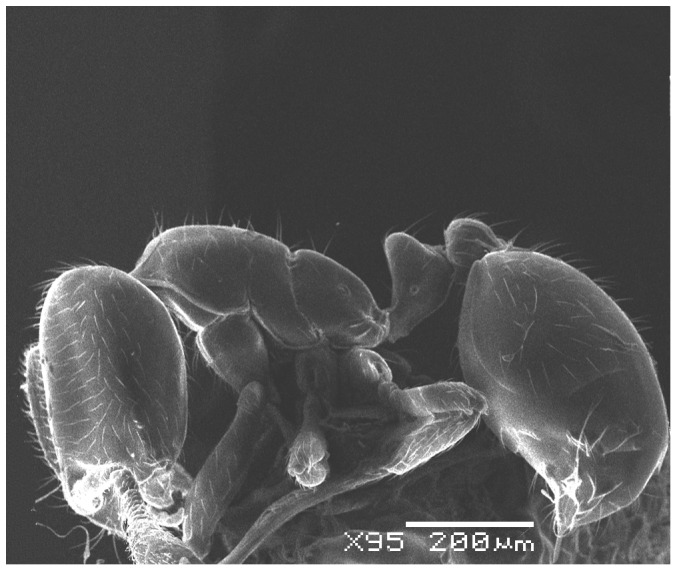
SEM of *Solenopsis saudiensis*, worker, body in profile.

**Figure 40:**
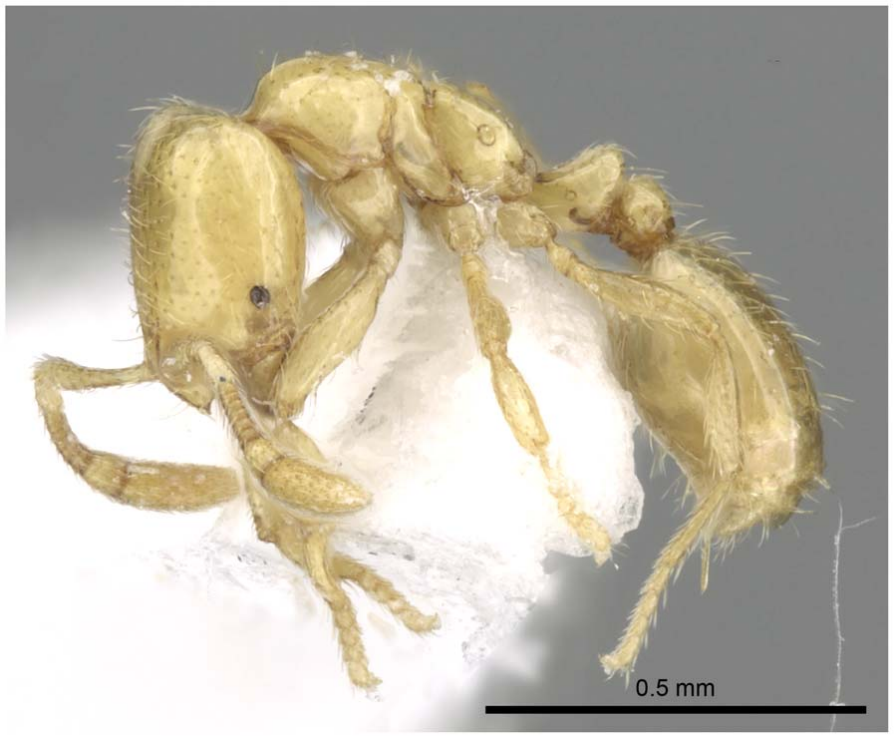
Automontage of *Solenopsis saudiensis*, worker, body in profile.

**Figure 41:**
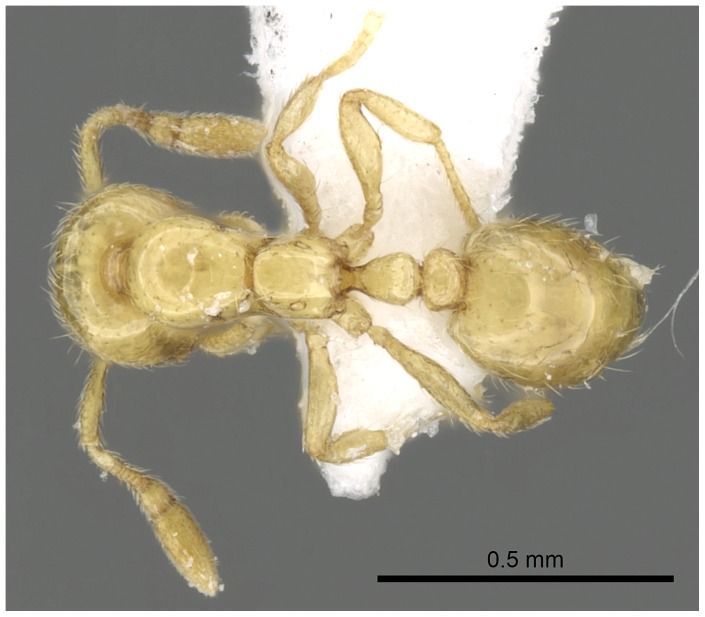
Automontage of *Solenopsis saudiensis*, worker, body in dorsal view.

**Figure 42:**
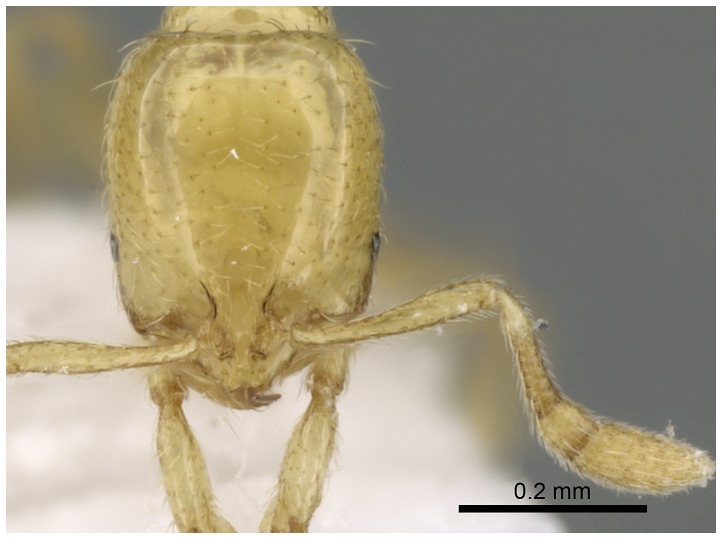
Automontage of *Solenopsis saudiensis*, worker, head in full-face view.

*Solenopsis saudiensis* Sharaf & Aldawood, 2011: 476. Holotype worker, SAUDI ARABIA: Riyadh, 24°43′N, 46°37′E, 9.VII.2009, 612 m *(Mostafa R. Sharaf & Abdulrahman S. Aldawood)* (KSMA) [examined]. Two paratype workers with same data as the holotype (KSMA) [examined]. 117 paratype workers, SAUDI ARABIA: Riyadh, Wadi Hanifa, 24°39′N, 46°36′E, 15.I.2010, 633m *(Mostafa R. Sharaf & Abdulrahman S. Aldawood)* (KSMA) [20 examined].

#### Holotype worker

TL1.3, HL 0.41, HW 0.31, SL 0.27, EL 0.02, ML 0.38, PL 0.10, PW 0.10, PPL 0.10, PPW 0.13, Indices: SI 87, CI 75.

#### Paratype workers

TL 1.2–1.3, HL 0.31–0.40, HW 0.30, SL0.21–0.27, EL 0.02, ML 0.31–0.35, PL 0.10, PW 0.10, PPL 0.10, PPW 0.10–0.11, Indices: SI 70–90, CI 75–97 (4 measured).

#### Diagnosis

Among *Solenopsis* species of the Arabian Peninsula, *S. saudiensis* is the only monomorphic species and can be immediately distinguished by its smaller size, TL 1.2–1.3 mm.

#### Description of worker

Head longer than broad in full face view; head dorsum smooth and shining with abundant scattered minute hair pits; eyes of two minute dark ommatidia; clypeus with a strongly impressed anterior margin and sharp carinae; anterior margin with a central pair of stout projecting teeth (0.02 mm), and a lateral pair of short, broad basal blunt teeth; mandibles with four reddish brown teeth, apical tooth is the largest, basal one is the smallest, second and third teeth subequal; antennae ten-segmented with a well-defined two-segmented club; scape fails to reach posterior margin of head; funicular segments two-seven about twice broader than long, with abundant, decumbent long yellow setae. Promesonotum with a smooth and uninterrupted profile; metanotal groove weakly but distinctly impressed; impression between mesopleuron and metapleuron faintly striate; propodeum short and low, with basal face making a continuous arc with the declivity, and a shallow dorsal longitudinal impression; spiracle is relatively large (0.02 mm diameter) and circular. Petiole as long as broad in dorsal view, in lateral view petiolar node high or slightly pointed; anterior peduncle with a distinct small ventral concave surface. Postpetiole about 1.3× broader than long; in profile node nearly hexagonal with a distinctly convex dorsal surface. All body parts are clothed with abundant, scattered, and moderately long yellow setae; cephalic pilosity short. Overall color uniform yellow.

#### Additional records

Rawdet Khoureim (Riyadh), 25°22′986″N, 47°16′712″E, 559m, 18.II.2012 (1).

#### Remarks

Queens and males are unknown.

### *Solenopsis sumara* Collingwood & Agosti, 1996 (Figs. 43–49)

**Figure 43:**
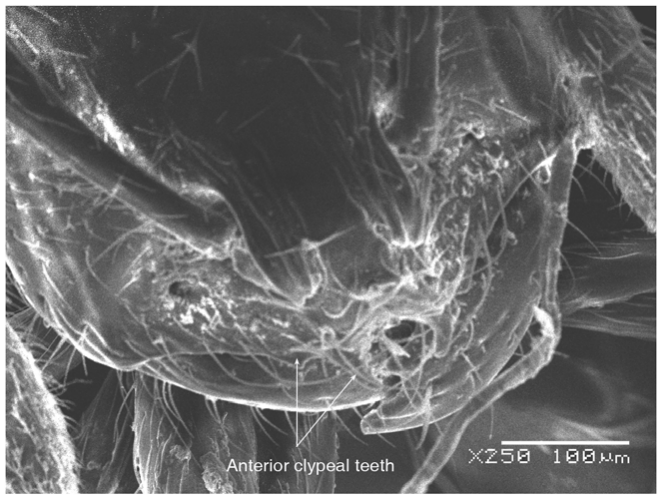
SEM of *Solenopsis sumara*, major worker, clypeus.

**Figure 44:**
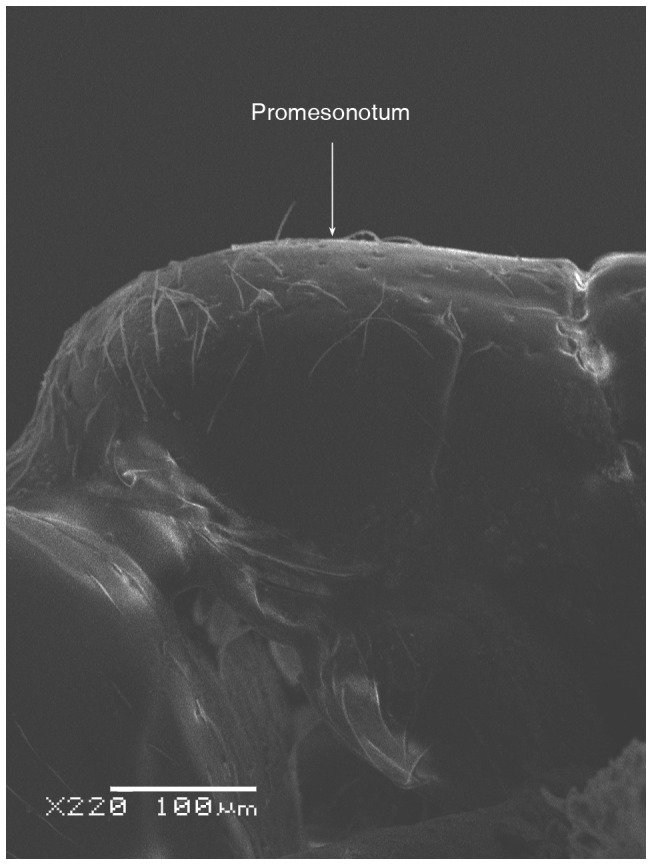
SEM of *Solenopsis sumara*, major worker, promesonotum in profile.

**Figure 45:**
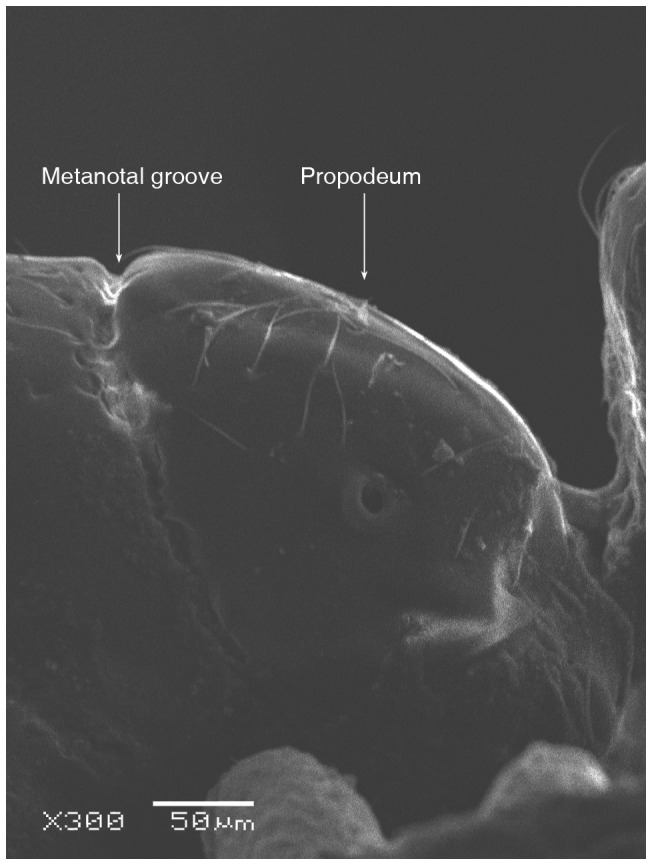
SEM of *Solenopsis sumara*, major worker, metanotal groove and propodeum in profile.

**Figure 46:**
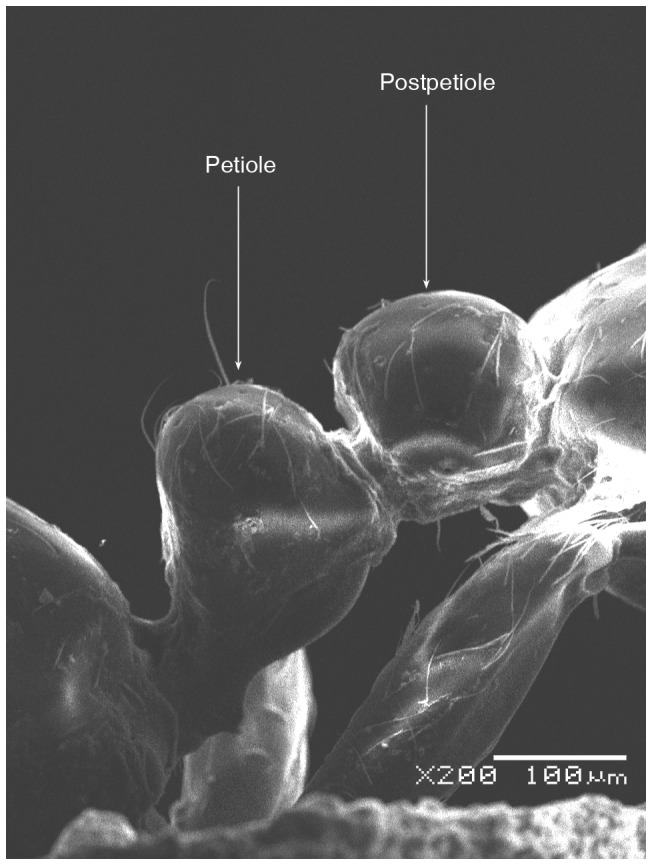
SEM of *Solenopsis sumara*, major worker, petiole and postpetiole in profile.

**Figure 47:**
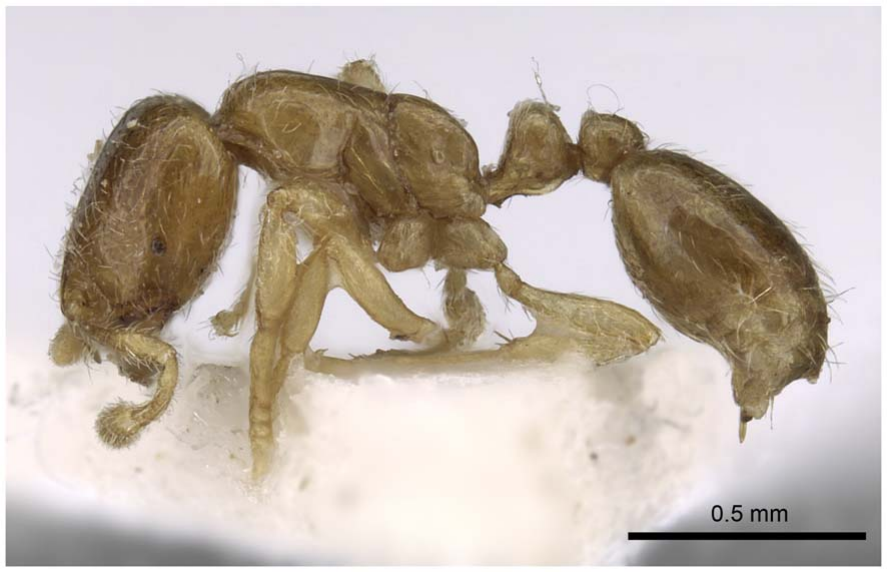
Automontage of *Solenopsis sumara*, major worker, body in profile.

**Figure 48:**
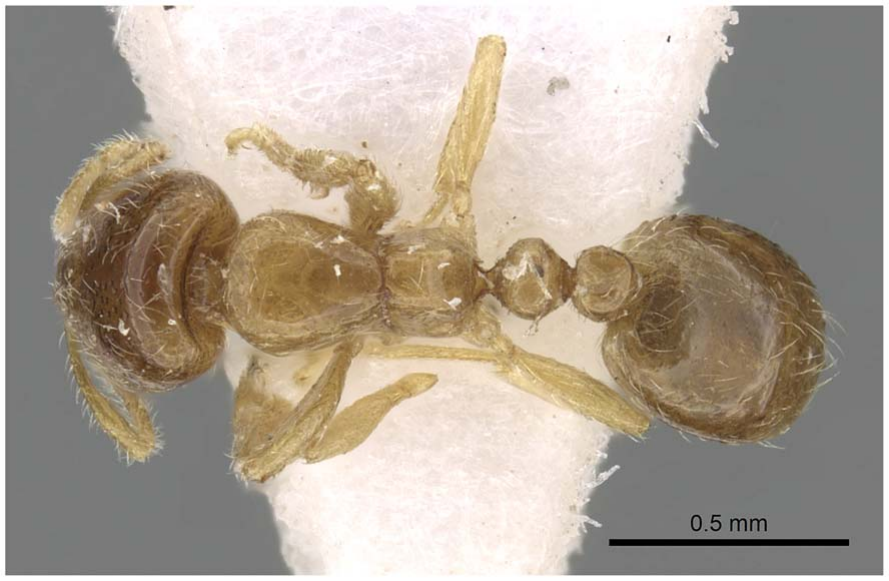
Automontage of *Solenopsis sumara*, major worker, body in dorsal view.

**Figure 49:**
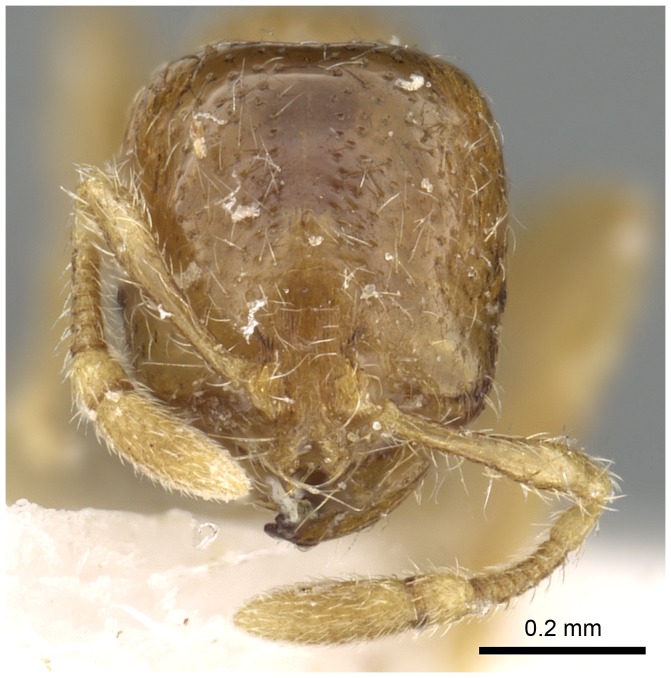
Automontage of *Solenopsis sumara*, major worker, head in full-face view.

*Solenopsis sumara* Collingwood & Agosti, 1996: 259. (2) major workers, YEMEN: Sumara Pass, 7.III.1993, 2800 m (sic 2500 in Collingwood & Agosti, 1996) *(C. A. Collingwood)* (WMLC) [examined, one of the workers is designated here as **lectotype** ].

#### Lectotype major worker

TL 2.37; HL 0.62; HW 0.50; SL 0.40; PRW 0.32; EL 0.04; ML 0.62; PL 0.20; PW 0.22; PPL 0.15; PPW 0.20; Indices: SI 80; CI 81.

#### Paralectotypes (major workers)

TL 1.68–2.48; HL 0.44–0.65; HW 0.36–0.55; SL 0.28–0.42; PRW 0.17–0.35; EL 0.04; ML 0.48–0.65; PL 0.15–0.20; PW 0.12–0.22; PPL 0.11–0.15; PPW 0.12–0.18; Indices: SI 71–83; CI 70–91 (5 measured).

#### Type

Lecotype major worker, same data of the type locality (WMLC).

#### Diagnosis

*Solenopsis sumara* is readily distinguished from its relative *zingibara* by the following characters: head subrectangular; frons without frontal striae; eyes tiny, with two ommatidia; petiolar node in profile massive, high and rounded, in dorsal view clearly broader than long.

#### Redescription of major worker

Head subrectangular with nearly parallel sides and narrower anteriorly than posteriorly and slightly but distinctly concave posterior margin; smooth and shining but with widely-spaced punctures; eyes minute, oval with two ommatidia, an upper larger one and a much smaller lower one (originally described as with two-three ommatidia); central clypeal teeth quite prominent and incurved with lateral teeth easily visible in full-face view (originally stated as hardly visible in dorsal view). Promesonotum flat; metanotal groove deep; basal face of propodeum forms a continuous curve with the declivity, which is much longer; spiracle circular. Petiole in profile with a massive and high rounded node and in dorsal view clearly broader than long or scarcely as long as broad; with a short peduncle. Postpetiole little broader than long or as long as broad, with node feebly convex or nearly flat. Pilosity abundant on the head but scarce on pronotum, mesonotum and gaster; propodeum bare or has only one pair of seta. Setae of scape slightly longer than maximum width of scape. Unicolorous dirty yellowish or yellowish brown, with lateral clypeal margins and mandibular teeth reddish yellow. General appearance smooth and shining.

#### Remarks

In the original description Collingwood and Agosti listed only three specimens, the holotype and two paratypes but no clearly labeled holotype could be found in WMLC and only two specimens with red labels [Sumara Pass, 7.iii.1993, 2800 m] are exist without clear mentioning they are paratypes. It is highly unlikely, that the authors collected exactly two workers for the species, and not more. To prove that we looked for any type materials in the alcohol preserved collection in WMLC and a small tube with six specimens that has the same type locality data was found. The mentioned tube has been identified by Cedric Collingwood (pers. comm.) as containing the type materials, therefore, we believe that the original type series is in existence. We designate a lecotype in this study to unequivocally ascertain the identity of the species *Solenopsis sumara*. Queens and males are unknown.

### *Solenopsis zingibara* Collingwood & Agosti, 1996

*Solenopsis zingibara* Collingwood & Agosti, 1996: 259. A Holotype worker and 7 paratype workers, YEMEN: Wadi near Zingibar, 21.III.1993 *(C. A. Collingwood)* [not in WMLC, presumably lost].

#### Measurements

TL 1.6–2.8; HL 0.50–0.70; HW 0.40–0.61; SL 0.35–0.40; SI 66; C I94

#### Diagnosis

*Solenopsis zingibara* is similar to *S. sumara*, both are the only yellowish brown species but the former can be readily recognized from the later by the following characters: head square; frons with six strong frontal striae; and eyes consist of three-four ommatidia; whereas *sumara* has a subrectangular head, frons without frontal striae; and eyes with only two ommatidia.

#### Worker

Description of Collingwood & Agosti [10]. Head rather square, only slightly longer than broad with gently curved sides and weakly concave posterior margin; eyes small with three-four ommatidia; central clypeal teeth prominent, lateral teeth slightly projecting and visible in dorsal view. In major workers head with six strong frontal striae and scattered coarse punctures. mesosoma and nodes with spaced punctulate sculpture; anterior pronotal edge slightly raised at the well-marked metanotal groove; propodeal dorsum shorter than declivity and obliquely rounded. Body and head pilosity sparse. Color yellowish brown and general aspect shining.

#### Remarks

The original paper (p. 301) stated all type material was deposited in the NHMB but no material of this species labeled as types can be found there (Daniel Burkhardt, Isabelle Pfander, Guy Knight & Tony Hunter, personal communication). Neither could one of us (MRS) find any specimens in the WMLC. Queens and males are unknown.

### Key to the Arabian Species of the Genus *Solenopsis* Including the Egyptian Species

1 Posterior margin of head in majors deeply emarginated with strongly convex temples and with a median furrow extending forward to frons (Fig. 27); propodeal dorsum of large and smaller workers with distinct dorsolateral carinae (Fig. 26) (Pantropical species) ……………………… ***geminata*** (Fabricius)

- Posterior margin of head straight or feebly concave but not deeply emarginated nor with median furrow (Figs. 1, 9, 14, 18); propodeal dorsum of large and smaller workers without carinae of any type………………………………………………2

2 Eyes with a single ommatidium ……………………………………………………3

- Eyes with more than one ommatidium (Figs. 7, 16, 35)….5

3 Head in lateral view with flat dorsal and ventral surfaces; head in full-face view with nearly parallel sides; propodeum profile distinctly angled at junction of dorsum and declivity (Egypt)…………………………………………… ***cooperi*** Donisthorpe

- Head in lateral view with convex dorsal and ventral surfaces; head in full-face view with sides nearly parallel or shallowly convex; propodeum profile with a smoothly rounded transition from dorsum to declivity………………………………………… 4

4 TL 1.30–1.71; head in full-face view with clearly parallel sides; eyes large; eye length 0.1 times head width or more; first gastral tergite dark brown; EL 0.04–0.05 (Egypt)………………………………………………………… ***occipitalis*** Santschi

- TL 2.0; head in full-face view with shallowly convex sides with rounded posterior corners; eyes smaller, eye length 0.04 times head width; whole gaster unicolorus yellow; EL 0.02 (Egypt)………………………………………………… ***kochi*** Finzi

5 Postpetiole with a distinct anteroventral flange seen in profile as a tooth-like projection (Figs. 6, 12) ………………………………………… 6

- Postpetiole without a distinct anteroventral projection (Figs. 25, 46)…………………………………………………………7

6 Head in lateral view appears narrow with a flat ventral surface; unicolorous yellow, cephalic dorsum and first gastral tergite yellow with brownish tint; eyes with seven ommatidia (Figs. 28–31)(Oman and United Arab Emirates) ………. ***omana*** Collingwood & Agosti

- head in lateral view appears thick with a flat dorsal surface and a distinctly convex ventral surface; head of major workers brownish yellow; eyes with 4–5 ommatidia (Figs. 1–21) (Saudi Arabia)……………………..……… ***elhawagryi*** **sp. n.**

7 Eyes with nine facets, the outer circle of facets enclosing a single relatively large facet (Egypt)…………………………………………………………***lou***

- Eyes with 2–4 facets…………………………………………………….8

8 Head rather square, only slightly longer than wide; eyes with only 3–4 ommatidia; in large workers, head with six strong frontal striae on the frons (Yemen)…………………………… ***zingibara*** Collingwood & Agosti

- Head subrectangular; eyes with only two ommatidia (Fig. 35); head normal, without the above mentioned six striae……………………………………9

9 Large dimorphic yellowish brown species, TL 1.50–2.48; head much broader HW 0.36–0.55 and much longer HL 0.44–0.68 (Fig. 49); propodeal dorsum a gradual flat slope which is slightly longer than declivity (Figs. 45, 47); petiole distinctly broader than long in dorsal view; petiole in profile with a massive and high rounded node (Fig. 46); area between frontal carinae finely longitudinally striated (Fig. 43) (Yemen)………………… ***sumara*** Collingwood & Agosti

- Smaller, monomorphic yellowish species, TL 1.2–1.3; head narrower HW 0.30–0.31 and shorter HL 0.31–0.41(Figs. 34, 42); propodeal dorsum making a continuous angle with the declivity, no distinction of propodeal angle (Figs. 37, 40); petiole as long as broad in dorsal view; petiolar node high and slightly pointed in lateral view (Fig. 38); area between frontal carinae smooth (Saudi Arabia)……………………… ***saudiensis*** Sharaf & Aldawood

## Discussion

Little is known about habitat and biology of Palaearctic and Arabian *Solenopsis*. Workers seem to build nests in the ground, sand mounds, and litter [4]. Some nest under stones, e g. *S. sumara*, *S. dentata* and *S. elhawagryi*. Or such as *S. cooperi* on Saloga and Ghazal islands in Nile River (Aswan, Egypt), nest in loose sandy soil close to bases of dead trees [13]. *Solenopsis saudiensis* nests under date palms, among decaying dropped fruit, directly under rocks or even inside discarded carpet. The latter species often coexists with *Tapinoma simrothi* Krausse that attends mealybugs [12]. *Solenopis geminata* is known to nest in open sunny areas of disturbed agricultural landscapes and around buildings, where nest mounds are defended aggressively by workers [7].

It appears that the *Solenopsis* in the Arabia is restricted to mid and lower elevations, usually below ca. 600 m. Despite repeated ant collections, there are only two records from the mountainous regions of southwestern Saudi Arabia and nearby Yemen. [10] reported *S. sumara* from Yemen, at 2500 m and, here, we report the new species S. *elhawagryi*. In Egypt, no *Solenopsis* were found in over 25 field trips to the high mountains of Sinai.

We hope our new descriptions and this revision will provide a foundation for future research into phylogeny, behavior, ecology, ecosystem structure and function, as well as systematic analyses of this fascinating, a seemingly important group of ants.
