# Correction: Ants of the Genus *Solenopsis* Westwood 1840 (Hymenoptera: Formicidae) in the Arabian Peninsula with Description of a New Species, *Solenopsis elhawagryi*

**DOI:** 10.1371/journal.pone.0101856

**Published:** 2014-06-26

**Authors:** The PLOS ONE Staff

There is an error in the Funding section of the article. The correct Funding statement is:

“This project was supported by the National Science, Technology and Innovation Plan (NSTIP), Strategic technologies program, grant number (11-BIO1974-02), the Kingdom of Saudi Arabia. The funders had no role in study design, data collection and analysis, decision to publish, or preparation of the manuscript.”
